# The Effect of CBM1 and Linker on the Oxidase, Peroxidase and Monooxygenase Activities of AA9 LPMOs: Insight into Their Correlation with the Nature of Reductants and Crystallinity of Celluloses

**DOI:** 10.3390/ijms252312616

**Published:** 2024-11-24

**Authors:** Xu Zhao, Fei Xie, Kaixiang Chen, Liangkun Long, Shaojun Ding

**Affiliations:** The Co-Innovation Center of Efficient Processing and Utilization of Forest Resources, Jiangsu Key Lab for the Chemistry & Utilization of Agricultural and Forest Biomass, College of Chemical Engineering, Nanjing Forestry University, Nanjing 210037, China; zhaox08ms@163.com (X.Z.); xiefei@njfu.edu.cn (F.X.); chenkaixiang@njfu.edu.cn (K.C.); longlk602@njfu.edu.cn (L.L.)

**Keywords:** lytic polysaccharide monooxygenases, linker, carbohydrate-binding module 1, electron donor, H_2_O_2_

## Abstract

This study explores the effect of carbohydrate-binding module 1 (CBM1) and the linker on the function of auxiliary activity 9 (AA9) lytic polysaccharide monooxygenases (LPMOs), with a particular focus on monooxygenase activity, using different crystallinity celluloses and electron donors. The tested C1/C4-oxidizing AA9 LPMOs exhibited higher oxidase and peroxidase activities compared to those of the C4-oxidizing AA9 LPMOs. While the presence of CBM1 promoted cellulose-binding affinity, it reduced the oxidase activity of modular AA9 LPMOs. The effect of CBM1 on peroxidase activity was variable and enzyme-specific. Its influence on monooxygenase activity was linked to the type of reductants and the crystallinity of celluloses. Overall, CBM1 improved the monooxygenase activity on high-, medium-, and low-crystallinity celluloses when ascorbic acid (AscA) was used as the electron donor. CBM1 also facilitated monooxygenase activity on high-crystallinity cellulose, but significantly inhibited monooxygenase activity on low-crystallinity cellulose when cellobiose dehydrogenase (CDH) was the electron donor. Linker truncation of *Nc*LOMO9C enhanced the cellulose-binding affinity but decreased both the oxidase and peroxidase activities. Linker truncation also impacted the monooxygenase activity in both the AscA-AA9 LPMO and *Af*CDH-AA9 LPMO systems, though its effect was less pronounced compared to that of CBM1. This work provides new insights into the role of the reductant type and cellulose crystallinity in the functionality of CBM1 and the linker in AA9 LPMOs.

## 1. Introduction

Lignocellulose is the most abundant renewable biomass on Earth. The enzymatic saccharification of cellulose and hemicellulose in lignocellulosic biomass to fermentable sugars is a critical step in the biorefinery process, promoting their conversion into value-added biofuels and biochemicals. However, enzymatic saccharification by conventional cellulases remains inefficient due to the high recalcitrance of crystalline cellulose [1,2]. Lytic polysaccharide monooxygenases (LPMOs) are copper ion-dependent oxidases first discovered in 2010, now classified into eight families (AA9-AA11 and AA13-AA17) within the auxiliary activities of the Carbohydrate-Active Enzymes (CAZY) database (http://www.cazy.org, accessed on 1 October 2024) [3,4,5]. Among these, the AA9 LPMOs catalyze the oxidative cleavage of glycosidic bonds at the C1 and/or C4 carbons in cellulose, increasing the enzymatic depolymerization of crystalline cellulose by providing more binding sites for glycoside hydrolase (GH). AA9 LPMOs are key components in modern commercial enzyme cocktails such as Cellic^®^CTec3 [6].

Compared to the hydrolytic action of glycoside hydrolases, the oxidative mechanism of LPMOs is more complex, requiring an electron donor to reduce Cu^2+^ to Cu^+^ and utilizing O_2_ or H_2_O_2_ as co-substrates to drive catalytic activity [7,8,9,10]. The electron donors can be small molecule reductants, such as ascorbic acid, or flavoproteins like cellobiose dehydrogenase (CDH) [11,12]. Different electron donors may vary in their capacity to deliver electrons and generate H_2_O_2_ during the reaction [13,14,15,16,17]. Additionally, the structural properties of the substrate, such as the surface area and crystallinity, may change during enzymatic saccharification, affecting the LPMO efficiency [18,19,20]. When oxygen is used as a co-substrate, the oxidative activity of LPMOs often involves multiple coupling and uncoupling pathways, with the balance between these pathways depending on the enzyme’s affinity for the substrate [19,21]. Strong enzyme–substrate binding favors the coupling pathway, promoting the oxidative cleavage of glycosidic bonds and preventing H_2_O_2_ formation, while weaker binding favors the uncoupling pathway, leading to enzyme-dependent H_2_O_2_ production [19]. The production of H_2_O_2_, whether through abiotic or enzyme-dependent oxidation, can accelerate oxidative reactions; however, excessive H_2_O_2_ results in the auto-oxidative inactivation of LPMOs [5,10,13,14]. In particular, when the enzyme does not bind to the substrate, active sites exposed to the solvent are highly susceptible to reactive oxygen species [10]. Consequently, LPMO monooxygenase activity and stability during lignocellulose degradation are influenced by both the electron donor and the substrate [5,9,12,13,18,19,20].

A significant portion of AA9 LPMOs are modular enzymes comprising a catalytic domain (CD) and a family 1 carbohydrate-binding module (CBM1), connected by a linker peptide [6,22,23]. Similarly to fungal glycoside hydrolases, the CBM1 in modular AA9 LPMOs serves as an auxiliary component for substrate binding, thereby influencing the enzymatic activity on insoluble lignocellulosic biomass [24,25,26,27,28]. Consequently, removing the CBM1 often results in decreased binding and oxidative activity, and can even alter the oxidative regioselectivity of AA9 LPMOs [29]. The linker peptide functions as a flexible spacer between the CD and CBM, allowing the two domains to move independently [18,22]. This peptide plays a key role in regulating the interaction between the CD and CBM domains and affects the overall conformation of multidomain cellulases [25,30]. Additionally, linkers have been shown to vary greatly in length, composition, and glycosylation depending on the specific structured domain [31]. Changes in the linker length and composition can impact the enzyme’s catalytic activity, adsorption efficiency, and thermal stability [18,32,33,34]. Furthermore, Hansson et al. found that the complete truncation of the linker peptide in *Hj*LPMO9A resulted in almost no enzyme expression [35]. These observations suggest that the linker region may play a crucial role in the functionality of modular AA9 LPMOs, similar to other carbohydrate-active enzymes.

Clarifying the functional relevance of the CBM1 and linker region to the activity and stability of modular LPMOs has gained significant interest in recent years [18,23,27,28,29]. Notably, in most studies, small organic molecules such as ascorbic acid (AscA) have been used as electron donors, but no report for enzyme partners. In the case of AscA, the reduction of Cu^2+^ to Cu^+^ can occur regardless of whether the LPMOs are bound to the substrate [3,5,18]. However, when enzyme partners like CDHs are used as electron donors, the strong binding of CDHs and/or AA9 LPMOs to the cellulosic substrate can hinder inter-protein electron transfer between CDH and AA9 LPMOs [36]. Since the presence of CBM1 significantly increases the binding affinity of modular AA9 LPMOs to cellulosic substrates [27,37,38], the hypothesis is that the effect of CBM1 on AA9 LPMO oxidative activity in the CDH-AA9 LPMO system may differ considerably from the effect observed when small organic molecules are used as electron donors. Currently, there is limited understanding of how the type and content of reductants, as well as the properties of cellulosic substrates, interact with the functionality of CBM1 and the linker in modular AA9 LPMOs.

In this study, the effects of CBM1 and the linker on the oxidase, peroxidase, and monooxygenase activities of C4-oxidizing and C1/C4-oxidizing AA9 LPMOs were systematically analyzed under varying conditions, including different types and concentrations of electron donors (AscA and AfCDH from *Aspergillus fumigatus*) and the crystallinity of celluloses. The primary objective of the study is to provide new insights into how the type of reductant and cellulose crystallinity affect the roles of CBM1 and the linker in the activity of AA9 LPMOs, which exhibit distinct modularity and oxidative regioselectivities. Our findings suggest that the effect of CBM1 and the linker on the oxidative activity of AA9 LPMOs is dependent on the nature of the reductant and the degree of cellulose crystallinity. Cellulolytic fungi contain multiple genes encoding AA9 LPMOs with different modularity and oxidative regioselectivities [12], while the availability and nature of electron donors and cellulosic substrates may vary during the biodegradation of lignocellulosic biomass [12,19,39,40]. This study suggests that AA9 LPMOs with and without CBM1 could be utilized to promote biomass degradation under various environmental conditions in vivo, as previously hypothesized [39,40]. This highlights the importance of the diversity of AA9 LPMOs in the biodegradation of lignocellulosic biomass in nature and the biorefinery process.

## 2. Results

### 2.1. Construction and Expression of the Wild-Type AA9 LPMOs and Variants

**Figure 1:**
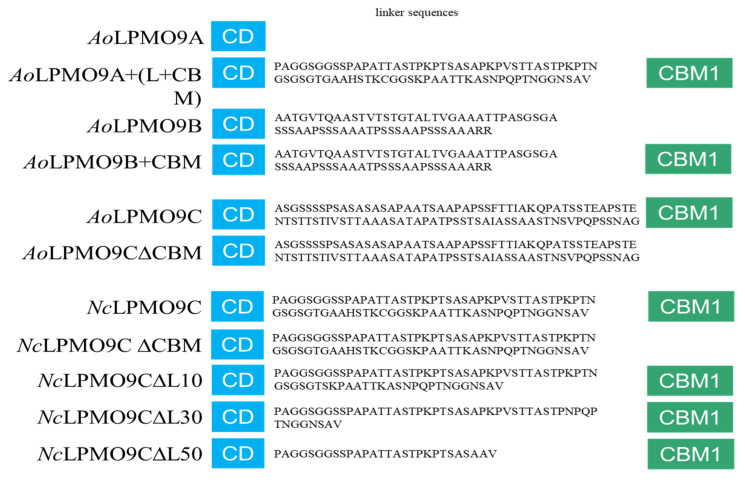
Schematic representation of the modular structures of *Ao*LPMO9A, 9B, 9C, and *Nc*LPMO9C and their variants. From top to bottom, they are *Ao*LPMO9A and its CBM1-fused variant; *Ao*LPMO9B and its CBM1-fused variant; *Ao*LPMO9C and its CBM1-truncated variant; *Nc*LPMO9C, its CBM1-truncated variant, and 10-, 30- and 50-amino-acid linker-truncated variants. The regioselectivity of wild-type AA9 LPMOs and variants was indicated in the right section of the figure. Abbreviations: CD, catalytic domain; CBM1, carbohydrate-binding module 1.

Based on the DNA sequences of wild-type *Ao*LPMO9A, 9B, 9C, and *Nc*LPMO9C from our previous studies [41], genes encoding variants including *Ao*LPMO9A+CBM, *Ao*LPMO9B+CBM, *Ao*LPMO9C∆CBM, *Nc*LPMO9C∆CBM, and *Nc*LPMO9C∆L10/∆L30/∆L50 were constructed and cloned into the pPICZαA vector (Figure 1). The linker regions near the structured domains in cellulases are relatively conserved and crucial for activity, while the middle segment of the linker is variable [34]. In addition, one potential N-glycosylation site (NGS) exists in the linker region. Therefore, the middle segment of the linker in *Nc*LPMO9C was truncated by 10, 30, or 50 amino acids. The detailed amino acid sequences of the linker regions of the individual wild-type AA9 LPMOs and variants are presented in Figure 1. All recombinant proteins, including wild-type *Ao*LPMO9A, 9B, 9C, and *Nc*LPMO9C, along with their corresponding variants, were overexpressed with a C-terminal 6xHis-tag in *Pichia pastoris* X33. The recombinant proteins were purified using Ni-chelating resin. The purity of the recombinant proteins was confirmed by SDS-PAGE, which showed that the size of the electrophoretic bands was larger than the theoretical molecular mass due to the presence of N-glycosylation (Figure S1). The peroxidase activity of the wild-type AA9 LPMOs and variants was tested as described in the experimental section, and the results confirmed that the recombinant enzymes were successfully expressed in their active forms.

### 2.2. Oxidase Activity Analysis on Basis of H_2_O_2_ Generation by AA9 LPMOs

**Figure 2:**
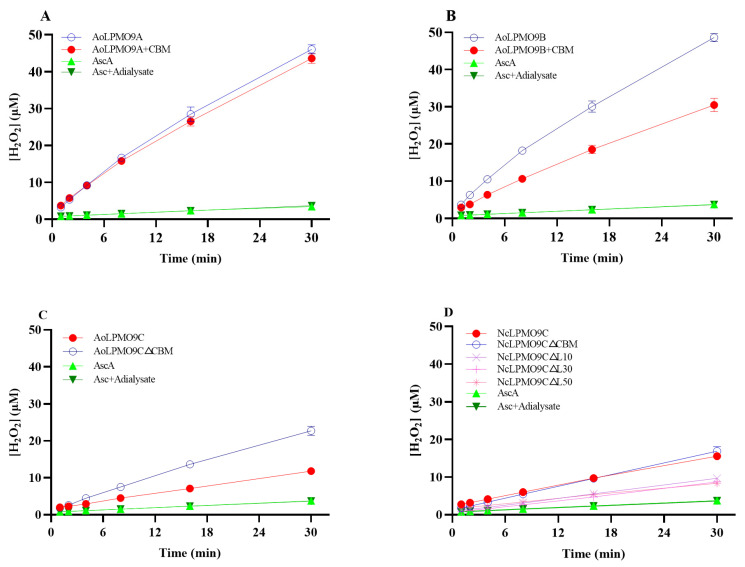
In situ generation of H_2_O_2_ by wild-type AA9 LPMOs and variants with 1 mM AscA. (**A**) *Ao*LPMO9A and its CBM1-fused variant (**B**) *Ao*LPMO9B and its CBM1-fused variant; (**C**) *Ao*LPMO9C and its CBM1-truncated variant; (**D**) *Nc*LPMO9C and its CBM1- or linker-truncated variants. Control reactions replacing AA9 LPMO with ultrapure water or third-dialysate were also carried out in parallel to evaluate the AA9 LPMO-independent H_2_O_2_ production or the influence of the residual free copper in the enzyme preparations on the H_2_O_2_ generation. The overlapping of curves of two controls means that the enzyme preparations contained negligible amounts of free copper. All assays were performed in triplicate.

It is well established that H_2_O_2_ can be produced as a result of the oxidase activity of LPMOs. In this study, the in situ generation of H_2_O_2_ by wild-type AA9 LPMOs and their variants in a reaction solution containing 1 mM AscA was measured to compare their oxidase activities [13,42]. As shown in Figure 2, H_2_O_2_ accumulated almost linearly over 30 min for all AA9 LPMOs. The initial H_2_O_2_ generation rates were calculated from these data and are presented in Table 1. The initial H_2_O_2_ generation rate (min^−1^) of all LPMOs containing CBM1 was relatively lower than that of the corresponding AA9 LPMOs without CBM1. Additionally, the truncated *Nc*LPMO9C∆L10/∆L30/∆L50 variants exhibited similar H_2_O_2_ generation rates, but these were much lower than that of wild-type *Nc*LPMO9C (Figure 2D). It is noteworthy that the C1/C4-oxidizing AA9 LPMOs displayed significantly higher H_2_O_2_ generation rates compared to the C4-oxidizing AA9 LPMOs.

### 2.3. Kinetic Constants of Wild-Type AA9 LPMOs and Variants for 2,6-DMP and H_2_O_2_

The kinetics of the LPMO peroxidase reaction for wild-type AA9 LPMOs and their variants using 2,6-DMP and H_2_O_2_ are presented in Figure S2 and Table 2. Notably, both C1/C4-oxidizing *Ao*LPMO9A and *Ao*LPMO9B exhibited significantly higher *k*_cat_ values for 2,6-DMP when using 5 mM H_2_O_2_ as a co-substrate, and for H_2_O_2_ when using 5 mM 2,6-DMP as the substrate, compared to the C4-oxidizing *Ao*LPMO9C and *Nc*LPMO9C (Table 2).

The fusion of CBM1 increased the *k*_cat_ values of *Ao*LPMO9A for both 2,6-DMP and H_2_O_2_, while it reduced the maximum reaction rate of *Ao*LPMO9B. Specifically, the *k*_cat_ of *Ao*LPMO9A+CBM for H_2_O_2_ was 11.5% higher than that of *Ao*LPMO9A, whereas the *k*_cat_ of *Ao*LPMO9B+CBM was 8.3% lower than that of *Ao*LPMO9B (Table 2). The removal of CBM1 from *Nc*LPMO9C also resulted in a reduction in peroxidase activity, with the *k*_cat_ value of *Nc*LPMO9C∆CBM for H_2_O_2_ being 22.3% lower than that of *Nc*LPMO9C with CBM1 (Table 2). However, removing CBM1 from *Ao*LPMO9C led to an increase in peroxidase activity, with the *k*_cat_ value of *Ao*LPMO9C∆CBM for H_2_O_2_ being 39.1% higher than that of *Ao*LPMO9C with CBM1 (Table 2). Furthermore, the truncation of the linker in *Nc*LPMO9C resulted in a 32.5–45.3% reduction in the peroxidase activity in terms of the *k*_cat_ (Table 2). Similar results were observed for 2,6-DMP. The inconsistent effects of CBM1 on the peroxidase activity suggest that alterations in the modular structure of the CD may be enzyme-specific. However, the decrease in both the peroxidase and oxidase activities (as noted in Section 2.1) in the linker-truncated variants of *Nc*LPMO9C indicates that adequate space between the two modules may be essential for the proper functionality of the CD.

Before their oxidative reaction, AA9 LPMOs are needed for the initial priming reduction of the resting-state LPMO-Cu(II) into the catalytically competent LPMO-Cu(I) form, then reduced LPMO can be re-oxidized by the O_2_ (oxidase) or H_2_O_2_ (peroxidase). In comparison, the oxidase activity of the AA9 LPMOs and their variants used in this study is much higher than their peroxidase activity (Table 1 and Table 2), indicating that the re-oxidation by O_2_ is more efficient than that by H_2_O_2_ in free forms.

### 2.4. Substrate Binding Affinity of Wild-Type AA9 LPMOs and Variants

The binding isotherms and properties of wild-type AA9 LPMOs and their variants on PASC-80% are shown in Figure S3 and Table 3. Compared to *Ao*LPMO9A and *Ao*LPMO9B, the maximum binding capacities (*B*_Max_) of *Ao*LPMO9A+CBM and *Ao*LPMO9B+CBM increased by 164% and 413%, respectively. Additionally, the dissociation constant (*K*_d_) decreased significantly, indicating that the presence of CBM1 greatly enhanced the substrate binding affinity of the modular C1/C4-oxidizing *Ao*LPMO9A+CBM and *Ao*LPMO9B+CBM (Table 3). Consequently, the truncation of CBM1 in *Ao*LPMO9C and *Nc*LPMO9C led to a reduction in the adsorption capacity on PASC-80% by 146% and 442%, respectively, and a corresponding decrease in the binding affinity, as indicated by the *K*_d_ values (Table 3). Interestingly, *Nc*LPMO9C∆L30 and *Nc*LPMO9C∆L50, with 30- and 50-amino-acid linker truncations, exhibited approximately twice the adsorption capacity of *Nc*LPMO9C. However, *Nc*LPMO9C∆L10 showed similar adsorption capacity to that of *Nc*LPMO9C.

### 2.5. Effects of CBM1 and Linker on Monooxygenase Activity of Wild-Type AA9 LPMOs and Variants Using AscA as the Electron Donor

**Figure 3:**
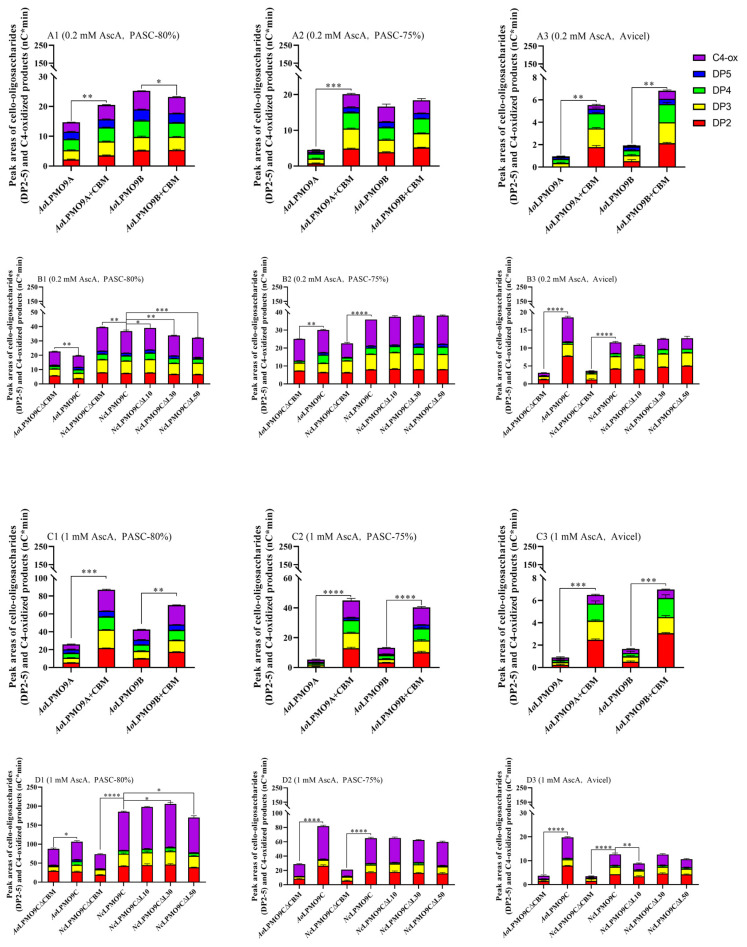
The sum of released cello-oligosaccharides and C4-oxidized products of different crystalline celluloses in AscA-AA9 LPMO system. (**A1**,**B1**) The 0.2 mM AscA and PASC-80%; (**A2**,**B2**) 0.2 mM AscA and PASC-75%; (**A3**,**B3**) 0.2 mM AscA and Avicel; (**C1**,**D1**) 1 mM AscA and PASC-80%; (**C2**,**D2**) 1 mM AscA and PASC-75%; (**C3**,**D3**) 1 mM AscA and Avicel. All assays were performed in triplicate. * *p* < 0.05, ** *p* < 0.01, *** *p* < 0.001, **** *p* < 0.0001.

The C4-oxidizing AA9 LPMOs (*Ao*LPMO9C, *Nc*LPMO9C, and their variants) produced native cello-oligos and C4-oxidized products, while the C1/C4-oxidizing AA9 LPMOs (*Ao*LPMO9A, *Ao*LPMO9B, and their variants) generated native cello-oligos along with C1- and C4-oxidized products. Since the C1-oxidized products overlapped with native cello-oligos with degrees of polymerization over 6 (DP6), the monooxygenase activity of the wild-type AA9 LPMOs and variants primed by AscA was quantified by summing the cello-oligos (DP2-5) and C4-oxidized product peak areas using HPAEC-PAD (Figure 3 and Figures S4–S6). When a low concentration of AscA (0.2 mM) was used as the electron donor and PASC-80% (low-crystallinity) as the cellulosic substrate, the CBM1-free AA9 LPMOs, including *Ao*LPMO9B, *Ao*LPMO9C∆CBM, and *Nc*LPMO9C∆CBM, produced slightly more cello-oligos and C4-oxidized products than their corresponding CBM1-containing enzymes, with the exception of *Ao*LPMO9A+CBM, which generated 35.2% more cello-oligos and C4-oxidized products than *Ao*LPMO9A (Figure 3A1,B1). However, when PASC-75% (medium-crystallinity) or Avicel (high-crystallinity) was used as the cellulosic substrate, all of the CBM1-containing AA9 LPMOs, including *Ao*LPMO9A+CBM, *Ao*LPMO9B+CBM, *Ao*LPMO9C, and *Nc*LPMO9C, produced significantly more cello-oligos and C4-oxidized products: 365%, 16%, 17%, and 62% or 548%, 313%, 493%, and 242% more, respectively, compared to their corresponding CBM1-free enzymes (Figure 3A2–B3). As the AscA concentration increased to 1.0 mM, all of the CBM1-containing AA9 LPMOs generated significantly more cello-oligos and C4-oxidized products from PASC-80% (20% to 230%), PASC-75% (193% to 700%), and Avicel (346% to 651%) compared to their CBM1-free counterparts (Figure 3C,D). These results indicate that, with few exceptions, the presence of CBM1 improves the monooxygenase activity of the AscA-AA9 LPMO system on celluloses with varying crystallinities. Notably, the contribution of CBM1 to the monooxygenase activity becomes more pronounced as the AscA concentration and cellulose crystallinity increase.

### 2.6. Effects of CBM1 and Linker on Monooxygenase Activity of Wild-Type AA9 LPMOs and Variants Using AfCDH as the Electron Donor

**Figure 4:**
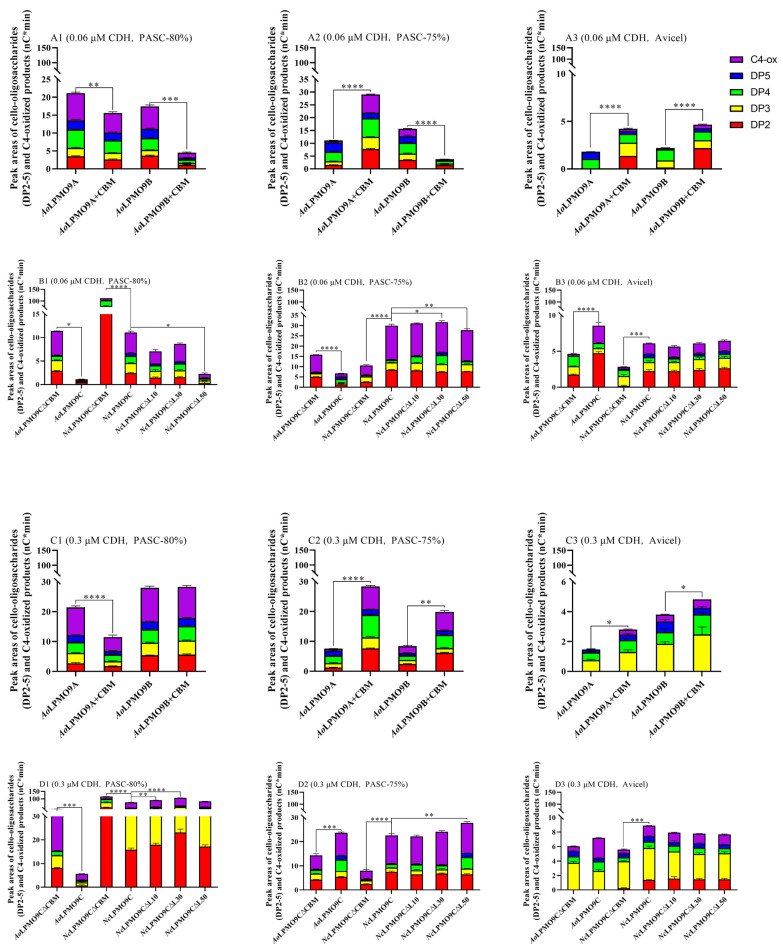
The sum of released cello-oligos and C4-oxidized products of different crystalline celluloses in CDH-AA9 LPMO system. (**A1**,**B1**) The 0.06 μM *Af*CDH and PASC-80%; (**A2**,**B2**) 0.06 μM *Af*CDH and PASC-75%; (**A3**,**B3**) 0.06 μM *Af*CDH and Avicel; (**C1**,**D1**) 0.3 μM *Af*CDH and PASC-80%; (**C2**,**D2**) 0.3 μM *Af*CDH and PASC-75%; (**C3**,**D3**) 0.3 μM *Af*CDH and Avicel. All assays were performed in triplicate. * *p* < 0.05, ** *p* < 0.01, *** *p* < 0.001, **** *p* < 0.0001.

When *Af*CDH was used as the electron donor, excessive lactose (10 mM) was added to the reaction system to ensure substrate availability for *Af*CDH. Similarly to using AscA as the electron donor, the monooxygenase activity of the wild-type AA9 LPMOs and their variants was quantified by the sum of the cello-oligo (DP2-5) and C4-oxidized product peak areas, measured using HPAEC-PAD (Figure 4 and Figures S7–S9). At a low concentration (0.06 µM) of *Af*CDH and using PASC-80% (low-crystallinity) as the cellulosic substrate, all of the CBM1-free AA9 LPMOs, including *Ao*LPMO9A, *Ao*LPMO9B, *Ao*LPMO9C∆CBM, and *Nc*LPMO9C∆CBM, produced significantly more cello-oligos and C4-oxidized products (38%, 311%, 930%, and 934% more, respectively) than their CBM1-containing counterparts (Figure 4A1,B1). When PASC-75% (medium-crystallinity) was used as the substrate, *Ao*LPMO9B and *Ao*LPMO9C∆CBM without CBM1 produced 293% and 145% more cello-oligos and C4-oxidized products, respectively, compared to their CBM1-containing variants. However, *Ao*LPMO9A+CBM and *Nc*LPMO9C with CBM1 produced 163% and 177% more cello-oligos and C4-oxidized products, respectively, than their CBM1-free counterparts (Figure 4A2,B2). When Avicel (high-crystallinity) was used as the substrate, all of the CBM1-containing AA9 LPMOs, including *Ao*LPMO9A+CBM, *Ao*LPMO9B+CBM, *Ao*LPMO9C, and *Nc*LPMO9C, produced 137%, 115%, 88%, and 126% more cello-oligos and C4-oxidized products, respectively, compared to their CBM1-free forms (Figure 4A3,4B3). At a higher concentration (0.3 µM) of *Af*CDH and using PASC-80% as the substrate, CBM1-free AA9 LPMOs, including *Ao*LPMO9A, *Ao*LPMO9C∆CBM, and *Nc*LPMO9C∆CBM, still produced significantly more cello-oligos and C4-oxidized products (87%, 445%, and 50%, respectively) than their CBM1-containing counterparts (Figure 4C1,D1). However, when PASC-75% or Avicel was used as the substrate, all of the CBM1-containing AA9 LPMOs, including *Ao*LPMO9A+CBM, *Ao*LPMO9B+CBM, *Ao*LPMO9C, and *Nc*LPMO9C, produced 287%, 147%, 61%, and 223%, or 81%, 23%, 8%, and 53% more cello-oligos and C4-oxidized products, respectively, compared to their CBM1-free forms (Figure 4C2–D3). These results indicate that the presence of CBM1 hindered the oxidative action of the *Af*CDH-AA9 LPMO system on low-crystallinity cellulosic substrates. However, the benefit of CBM1 became evident when medium- or high-crystallinity substrates were used, particularly at higher concentrations of *Af*CDH.

The truncation of the linker had a slight to moderate effect on the monooxygenase activity of *Nc*LPMO9C, depending on the electron donor and the crystallinity of the cellulose. When AscA was used as the electron donor, a slight difference in the monooxygenase activity was observed between *Nc*LPMO9C∆L10/∆L30/∆L50 and *Nc*LPMO9C, which was influenced by changes in the electron donor concentration and cellulose crystallinity (Figure 3B3,D3). When *Af*CDH (0.06 µM) was used as the electron donor, the monooxygenase activity of *Nc*LPMO9C∆L10/∆L30/∆L50 was similar to that of *Nc*LPMO9C on PASC-75% and Avicel. However, on PASC-80%, the monooxygenase activity of *Nc*LPMO9C∆L10/∆L30/∆L50 was 35%, 23%, and 79% lower, respectively, compared to *Nc*LPMO9C (Figure 4B). At a higher concentration of *Af*CDH (0.3 µM), slight positive or negative effects on the monooxygenase activity of *Nc*LPMO9C∆L10/∆L30/∆L50 were observed, depending on the cellulose crystallinity (Figure 4D).

### 2.7. Changes in H_2_O_2_ Concentration During Substrate-Oxidation Process of Wild-Type AA9 LPMOs and Variants Using AscA or AfCDH as Electron Donor

**Figure 5:**
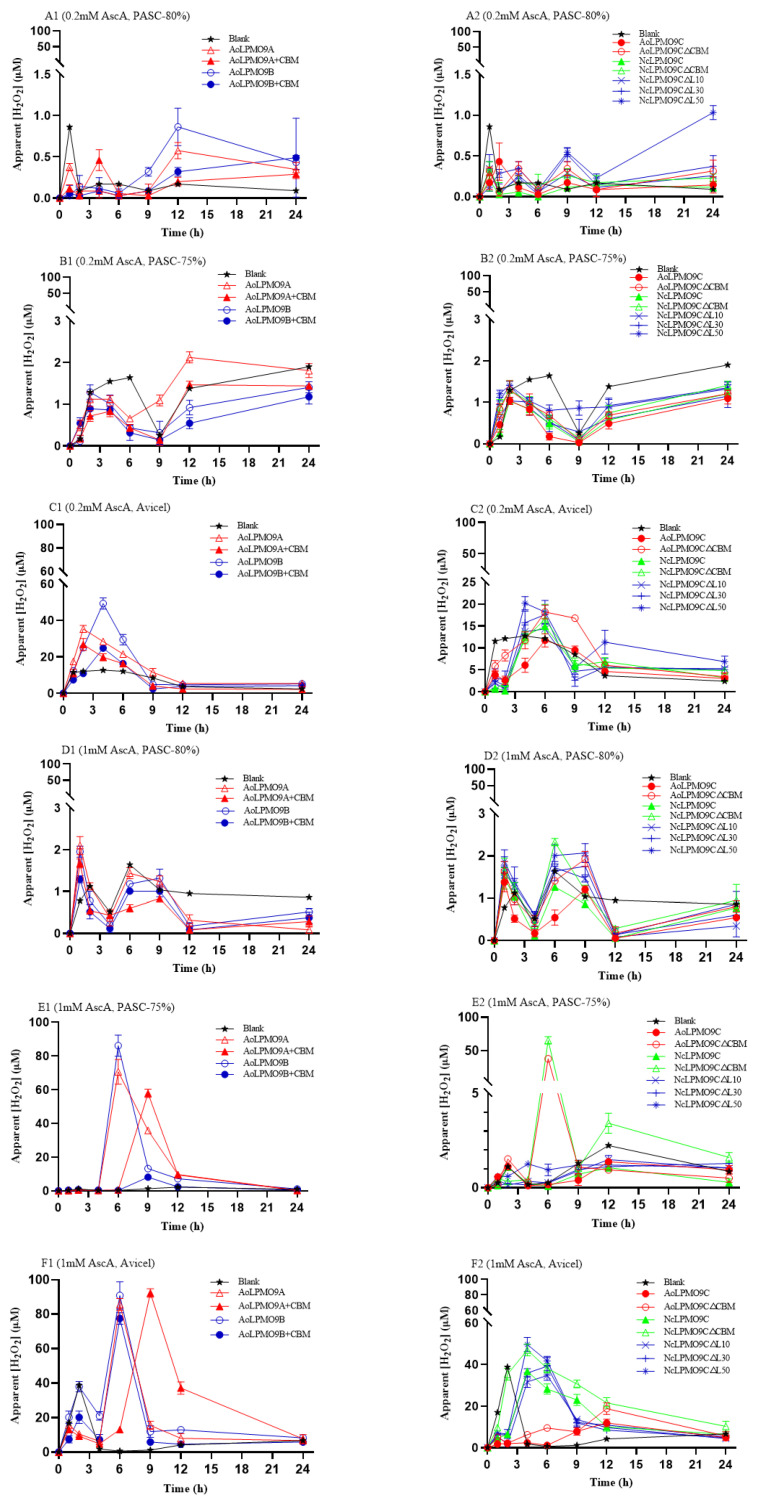
The change in the apparent H_2_O_2_ concentration during substrate-oxidation process of wild-type AA9 LPMOs and variants in AscA-AA9 LPMO system. (**A1**,**A2**) The 0.2 mM AscA and PASC-80%; (**B1**,**B2**) 0.2 mM AscA and PASC-75%; (**C1**,**C2**) 0.2 mM AscA and Avicel; (**D1**,**D2**) 1 mM AscA and PASC-80%; (**E1**,**E2**) 1 mM AscA and PASC-75%; (**F1**,**F2**) 1 mM AscA and Avicel. The control reactions performed by replacing AA9 LPMO with ultrapure water were also carried out in parallel to evaluate the AA9 LPMO-independent H_2_O_2_ production. All assays were performed in triplicate.

**Figure 6:**
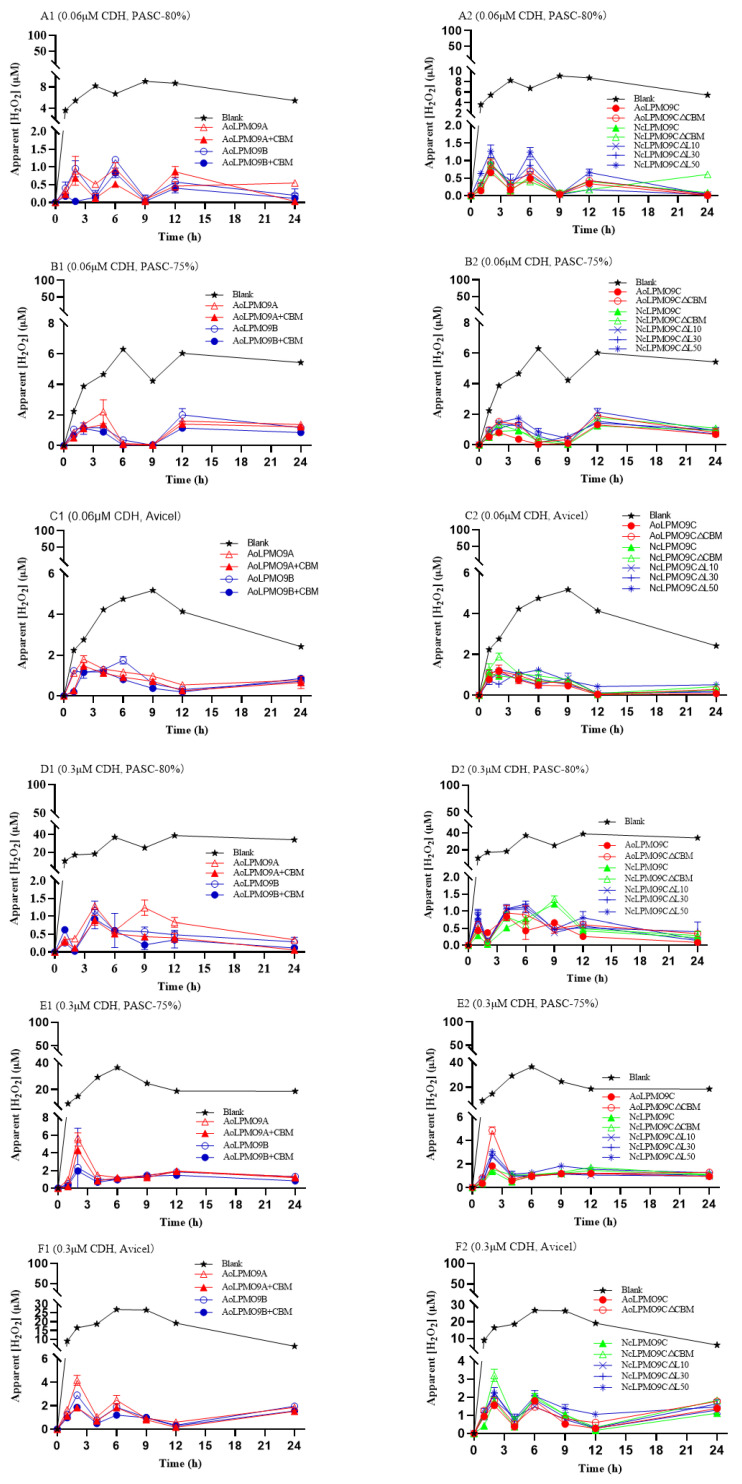
The change in the apparent H_2_O_2_ concentration during substrate-oxidation process of wild-type AA9 LPMOs and variants in *Af*CDH-AA9 LPMO system. (**A1**,**A2**) The 0.06 μM *Af*CDH and PASC-80%; (**B1**,**B2**) 0.06 μM *Af*CDH and PASC-75%; (**C1**,**C2**) 0.06 μM *Af*CDH and Avicel; (**D1**,**D2**) 0.3 μM *Af*CDH and PASC-80%; (**E1**,**E2**) 0.3 μM *Af*CDH and PASC-75%; (**F1**,**F2**) 0.3 μM *Af*CDH and Avicel. The control reactions performed by replacing AA9 LPMO with ultrapure water were also carried out in parallel to evaluate the AA9 LPMO-independent H_2_O_2_ production. All assays were performed in triplicate.

The H_2_O_2_ concentration in the reaction mixture during the substrate-oxidation process of wild-type AA9 LPMOs and their variants, using either AscA or *Af*CDH as electron donors, was measured at 3 h intervals using the modified HRP/Amplex Red assay. The results are shown in Figure 5 and Figure 6. When using a low concentration (0.2 mM) of AscA as the electron donor, despite some fluctuations, the apparent H_2_O_2_ concentration remained relatively low (less than 2.0 µM) for all enzymes during the substrate-oxidation processes with PASC-80% or PASC-75% as the cellulosic substrates (Figure 5A,B). However, a significant increase in the H_2_O_2_ concentration was observed when using Avicel as the substrate (Figure 5C). At a higher concentration of AscA (1.0 mM), the apparent H_2_O_2_ concentration in the reaction with PASC-80% as the substrate remained relatively low (less than 2.0 µM), but a notable increase in the H_2_O_2_ concentration was detected with both PASC-80% and PASC-75% as substrates (Figure 5D–F). It is worth noting that the apparent H_2_O_2_ concentration in reactions involving AA9 LPMOs without CBM1 was generally higher, or their peak H_2_O_2_ concentrations appeared earlier, than in reactions involving the AA9 LPMOs with CBM1.

When *Af*CDH was used as the electron donor at either 0.06 µM or 0.3 µM, the apparent H_2_O_2_ concentration in all the reaction mixtures remained relatively low and stable (less than 2 µM at 0.06 µM, and less than 6 µM at 0.3 µM *Af*CDH) during the substrate-oxidation process (Figure 6). As expected, high H_2_O_2_ concentrations were detected in the blank control, with maximum concentrations reaching 5–8 µM (Figure 6A–C) or 30–40 µM (Figure 6D–F) depending on the *Af*CDH dosage. Consistent with the results using AscA as the electron donor, the apparent H_2_O_2_ concentrations in the reactions with the AA9 LPMOs without CBM1 were typically higher than in those with CBM1.

## 3. Discussion

In this study, eleven wild-type AA9 LPMOs and their variants, with different modular structures and oxidative regioselectivities (including C4-oxidizing and C1/C4-oxidizing), were constructed through CBM1 fusion or CBM1 and linker truncation. It was observed that the oxidase and peroxidase activities of the C1/C4-oxidizing *Ao*LPMO9A and *Ao*LPMO9B were significantly higher than those of the C4-oxidizing *Ao*LPMO9C and *Nc*LPMO9C. This notable difference in oxidase and peroxidase activities between AA9 LPMOs with varying oxidative regioselectivities or from different sources has also been reported in previous studies [43,44]. For instance, the C1-oxidizing *Ma*LPMO9K and *Ms*LPMO3 from *Talaromyces sedimenticola* and *Morchella sextelata*, respectively, displayed peroxidase activities of 265 and 443 U/mg on 2,6-DMP [43,44], while the C1/C4-oxidizing AA9 LPMO cPMO2 from composting exhibited only 14.71 U/mg of peroxidase activity on the same substrate [45]. Sun et al. [46] reported that the C1-oxidizing *Mt*LPMO9B and *Mt*LPMO9I showed much higher oxidase activity than the C4-oxidizing *Mt*LPMO9E, and C1/C4-oxidizing *Mt*LPMO9H from *Myceliophthora thermophila* (*Mt*LPMO9s). However, the C1-oxidizing *Nc*LPMO9F had significantly lower oxidase activity compared to the C4-oxidizing *Nc*LPMO9C and the C1/C4-oxidizing *Nc*LPMO9M from *N. crassa*, suggesting that the oxidase and peroxidase activities of LPMOs may be influenced by the intrinsic catalytic potential of their catalytic domains. The substantial differences in the oxidase and peroxidase activities between the tested C1/C4-oxidizing and C4-oxidizing AA9 LPMOs may be largely attributed to variations in their copper active sites.

The presence of CBM1 reduced the oxidase activity of the modular AA9 LPMOs, consistent with previous findings [42]. However, the effect of CBM1 on the peroxidase activity varied and appeared to be enzyme-specific. The addition of native or fused CBM1 to both the wild-type AA9 LPMOs and their variants significantly increased their adsorption capacity on cellulosic substrates. These results align with earlier studies on cellulose-active AA9 and AA10 LPMOs, such as *Pa*LPMO9H, *Hj*LPMO9A, and *Sc*LPMO10C, as well as cellulolytic enzymes like cellobiohydrolase and endo-β-1,4-glucanase [22,23,24,25,29]. While the role of CBM1 in substrate binding for modular LPMOs is well documented, few studies have examined the influence of the linker region, specifically its length and N-glycosylation sites, on the binding properties of these enzymes. Srivastava et al. reported that truncating the linker in *Bc*LPMO9C led to a 1.5- to 7-fold reduction in the enzyme adsorption capacity [18]. In this study, 10, 30, and 50 amino acids from the middle of the linker region of *Nc*LPMO9C were sequentially truncated. The results found that *Nc*LPMO9C∆10 had a similar adsorption capacity to that of *Nc*LPMO9C, while *Nc*LPMO9C∆30 and *Nc*LPMO9C∆50 showed approximately twice the adsorption capacity of that of *Nc*LPMO9C. A NetNGlyc 1.0 analysis (http://www.cbs.dtu.dk/services/NetNGlyc/, accessed on 1 October 2024) revealed that *Nc*LPMO9C contains two potential N-glycosylation sites: one located in the CD region and the other in the linker region. The two N-glycosylation sites were retained in *Nc*LPMO9C∆10, but the linker region glycosylation site was removed in *Nc*LPMO9C∆30 and *Nc*LPMO9C∆50. Previous studies have suggested that the removal of glycans in the linker region of fungal cellulases can reduce their binding affinity to cellulose surfaces [25,32]. However, contrary to these earlier findings, the current study suggests that the significant increase in the adsorption capacity observed in *Nc*LPMO9C∆30 and *Nc*LPMO9C∆50 may result from the truncation of a potential N-glycosylation site in their linker region.

It is worth noting that *Nc*LPMO9C has a 79-amino-acid linker, which is considerably longer than the 55-amino-acid linker in *Bc*LPMO9C. Additionally, the two enzymes differ in the amino acid composition of their linkers. Therefore, beyond glycosylation, the differing effects of linker truncation may also be attributed to variations in the linker length, amino acid content, and the stiffness or flexibility of the linkers in *Bc*LPMO9C and *Nc*LPMO9C [18,31,32]. Furthermore, the linker truncation in *Nc*LPMO9C not only increased the binding affinity but also reduced the oxidase and peroxidase activities, suggesting that sufficient space between the two modules is likely critical for the catalytic function of the enzyme’s domain. This study indicates that the linker region plays an enzyme-specific role in the substrate binding and catalytic activity of the AA9 LPMOs. Consequently, the targeted modification of the linker region could alter the enzyme’s performance, as has been demonstrated in cellulase engineering [33,34].

AscA is commonly used as a small molecule reductant in most laboratory experiments; however, a variety of fungal redox enzymes are also crucial for priming AA9 LPMO reactions in nature [11,12]. Since CDH is typically co-secreted with other carbohydrate-active enzymes, including glycoside hydrolases and auxiliary activity family enzymes, it is considered the native reducing partner of AA9 LPMOs in fungal saprotrophs [11,12,47,48]. The wild-type AA9 LPMOs and variants used in this study originate from ascomycetous fungi, so *Af*CDH from the ascomycetous fungus *A. fumigatus* was chosen for this research. This study investigated the effects of CBM1 and linker regions on the apparent monooxygenase activity of wild-type AA9 LPMOs and their variants in both the AscA-AA9 LPMO and *Af*CDH-AA9 LPMO systems over an extended reaction period (24 h), with varying concentrations of reductants and different cellulose crystallinities. The presence of native or fused CBM1 had a significant effect on the apparent monooxygenase activity of the wild-type AA9 LPMOs and variants. However, this impact was highly dependent on the reductant type and the crystallinity of the cellulose. Generally, with medium- and high-crystallinity cellulose (such as PASC-75% and Avicel) and a high concentration of electron donors (1 mM of AscA or 0.3 µM of *Af*CDH), CBM1 greatly increased the apparent monooxygenase activity of the modular AA9 LPMOs in both the AscA-AA9 LPMO and *Af*CDH-AA9 LPMO systems, with a few exceptions. However, when using low-crystallinity cellulose (PASC-80%) as a substrate, the effects of CBM1 varied between the two systems. In the AscA (1.0 mM)-AA9 LPMO system, the CBM1-containing AA9 LPMOs released more cello-oligos and C4-oxidized products than their CBM1-free counterparts when using PASC-80% (Figure 3). At a lower concentration of AscA (0.2 mM), the contribution of CBM1 to the apparent monooxygenase activity became less significant, and in some cases, even a slight negative effect was observed. In contrast, in the *Af*CDH (0.3 µM)-AA9 LPMO system, the CBM1-containing enzymes released significantly fewer cello-oligos and C4-oxidized products than their CBM1-free counterparts from PASC-80% (low-crystallinity cellulose) (Figure 4). At a lower concentration (0.06 µM) of *Af*CDH, the inhibitory effect of CBM1 became even more pronounced, not only for all of the CBM1-containing AA9 LPMOs on PASC-80% but also for some CBM1-containing AA9 LPMOs (such as *Ao*LPMO9B+CBM and *Ao*LPMO9C) on PASC-75% (medium-crystallinity cellulose).

As mentioned earlier, the H_2_O_2_ concentration plays a crucial role in both the stability and oxidative reactions of LPMOs. In turn, changes in the H_2_O_2_ concentration during the substrate-oxidation process can indicate the state of LPMO activity [5,16,47,49]. Since the oxidase activities of AA9 LPMOs and their variants are significantly higher than their peroxidase activities (Table 1 and Table 2), the H_2_O_2_ generation is more efficient than the H_2_O_2_ consumption in the absence of a cellulosic substrate. On the other hand, the presence of CBM1 reduced the oxidase activity of the modular AA9 LPMOs, decreasing the H_2_O_2_ generation efficiency. So, to elucidate the differing effects of CBM1 on the apparent monooxygenase activities between the AscA-AA9 LPMO and *Af*CDH-AA9 LPMO systems, the H_2_O_2_ concentrations were monitored throughout the substrate-oxidation process. In the AscA-AA9 LPMO system, the H_2_O_2_ concentration was more readily influenced by both the concentration of AscA and the crystallinity of the cellulose compared to in the *Af*CDH-AA9 LPMO system. A relatively low and stable H_2_O_2_ concentration was only maintained when using a low concentration of AscA (0.2 mM) or low-crystallinity cellulose (PASC-80%) during the substrate-oxidation process. Otherwise, excessive H_2_O_2_ accumulation occurred, particularly for the CBM1-free AA9 LPMOs. This was observed in reactions with 0.2 mM AscA and Avicel (high-crystallinity cellulose), as well as with 1.0 mM AscA and either Avicel or PASC-75% (medium-crystallinity cellulose), leading to self-inactivation and the cessation of monooxygenase activity in the AA9 LPMOs lacking CBM1. In such conditions, the presence of CBM1 helped prevent enzyme self-inactivation by reducing the H_2_O_2_ concentration, likely due to the stronger substrate-binding affinity and lower oxidase activity of the CBM1-containing AA9 LPMOs. This may explain why the CBM1-containing AA9 LPMOs produced more cello-oligos and C4-oxidized products than their CBM1-free counterparts in the AscA-AA9 LPMO system. In the *Af*CDH-AA9 LPMO system, the H_2_O_2_ concentration remained relatively low and stable during the substrate-oxidation process. However, the strong substrate-binding affinity provided by CBM1 could hinder inter-protein electron transfer, particularly in the low-crystallinity PASC-80% system, where more accessible surface area is available for AA9 LPMO binding [20]. Consequently, CBM1 had a significant inhibitory effect in the *Af*CDH-AA9 LPMO system when using low-crystallinity PASC-80% as the substrate. This effect was further exacerbated when low concentrations of *Af*CDH were used, where both the electron transfer and H_2_O_2_ availability became rate-limiting for the apparent monooxygenase activity. To the best of our knowledge, this study represents the first investigation into the changes in the H_2_O_2_ concentration during the cellulose oxidation process by AA9 LPMOs using different reductants. This work may deepen our understanding of how the nature of reductants and cellulose crystallinity influence the roles of CBM1 and the linker in AA9 LPMOs. It is important to note that this study focuses specifically on the effects of ascorbic acid (AscA) and cellobiose dehydrogenase (*Af*CDH) as electron donors, without extending to a broader spectrum of reductants. Different reductants may exhibit varying rates of H_2_O_2_ generation and electron transfer, potentially affecting the LPMO activity and the role of CBM1 in distinct ways. Future studies could investigate a wider range of reductants to develop a more comprehensive understanding of CBM1’s functionality.

Overall, it is evident that CBM1 promotes the binding affinity of AA9 LPMOs to cellulose but reduces the rate of H_2_O_2_ production in oxidase reactions, as previously reported [42]. CBM1 and the linker region also influence the monooxygenase activities of AA9 LPMOs; however, the nature and extent of these effects are highly dependent on the type of reductant and cellulose used [8,23,37,50,51,52]. The presence of CBM1 appears crucial for the oxidative attack of AA9 LPMOs on high-crystallinity cellulose, regardless of whether AscA or CDH is used as the electron donor. Specifically, CDH may preferentially activate the oxidative activity of CBM1-free AA9 LPMOs on low-crystallinity cellulose, while small molecule reductants may more effectively fuel CBM1-containing AA9 LPMOs on high-crystallinity cellulose during the natural oxidative cleavage of cellulose. Notably, a few exceptions were observed, particularly at low electron donor concentrations and with low-crystallinity cellulose for certain AA9 LPMOs. These inconsistencies may reflect variations in intrinsic and enzyme-specific characteristics among different AA9 LPMOs [42,53,54]. Developing cost-effective cellulose-degrading enzymes is essential for the economical production of biofuels and biochemicals from lignocellulosic biomass. This process requires not only the involvement of multiple glycoside hydrolases but also several auxiliary activity family enzymes, including various LPMOs and components or methods that supply electron donors [4]. Our findings suggest that combining a small reducing agent with the enzyme partner CDH is crucial for activating various AA9 LPMOs. This approach can optimally utilize the potential of multiple AA9 LPMOs in enzymatic saccharification, thereby lowering the overall saccharification cost in industrial bioprocessing. However, to simplify the reaction system, this study employed pure celluloses instead of pretreated lignocellulosic biomass. It is important to note that real pretreated lignocellulosic biomass contains cellulose, hemicellulose, and residual lignin. Additionally, depending on the pretreatment method, various small phenolic compounds and H_2_O_2_ are generated [55]. These phenolic compounds may act as small reductants to initiate AA9 LPMO reactions but could also inhibit cellulase and hemicellulase activities. Future research could investigate the functional mechanisms, adaptive characteristics, and performance of the LPMO system under a combined glycoside hydrolase-oxidoreductase framework in diverse conditions to better understand its potential in practical applications involving pretreated lignocellulosic biomass.

Although this study highlights that CBM1 affects both binding affinity and electron transfer, the precise molecular mechanisms remain unclear. Furthermore, this research does not explore the effects of other reaction conditions, such as the temperature and pH, on the CBM1 functionality. These factors might influence CBM1’s binding affinity to cellulose, thereby impacting the oxidative activity. Future studies could examine how CBM1 modulates electron transfer rates, contributes to the generation of oxidative reaction products, and performs in synergistic interactions within multimodular structures. A deeper understanding of CBM1’s role under various conditions could provide valuable insights into the applicability of multiple AA9 LPMOs with varying modularity and oxidative regioselectivities in both natural environments and industrial settings.

## 4. Materials and Methods

### 4.1. Strains and Plasmids

*Escherichia coli* strain TOP10 was used for vector construction and propagation. *Pichia pastoris* strain X33 and the pPICZαA plasmid were utilized as the host and vector for the expression of wild-type AA9 LPMOs and their variants. All media and protocols followed the standard instructions provided in the EasySelect Pichia Expression kit (Invitrogen, Carlsbad, CA, USA).

### 4.2. Cellulosic Substrate Preparation

PASC (phosphoric acid-swollen cellulose, designated as PASC-75% and PASC-80%, respectively) with varying degrees of crystallinity was prepared from Avicel^®^ PH-101 (Fluka, Buchs, Switzerland) by swelling with different concentrations of phosphoric acid (75% and 80% (*v*/*v*), respectively) following the method of Chen et al. [41]. The specific crystallinity index (CrI) of Avicel^®^ PH-101, PASC-75%, and PASC-80% was 80.2%, 74.6%, and 60.1%, respectively, as determined in our previous experiments described by Chen et al. [41]. Thus, Avicel, PASC-75%, and PASC-80% represent high-, medium-, and low-crystallinity celluloses in this study.

In addition, Avicel has a rod-like shape, but after treatment with 75% and 80% phosphoric acid, the cellulose swelled and partially dissolved, resulting in a structural change to a sheet-like form with hollow regions. This indicated that the surface areas of PASC-75% and PASC-80% were greater than that of Avicel [41].

### 4.3. Construction of Variants

To investigate the effects of CBM1 and the linker on enzyme activity, seven new AA9 LPMO variants with different modular structures were constructed from previously characterized wild-type *Ao*LPMO9A (GenBank accession no. XP001821292), *Ao*LPMO9B (GenBank accession no. XP001727159), *Ao*LPMO9C (GenBank accession no. XP023088996) from *Aspergillus oryzae*, and *Nc*LPMO9C (GenBank accession no. XP965598) from *Neurospora crassa* [41]. These variants included (1) *Ao*LPMO9A+CBM and *Ao*LPMO9B+CBM, where CBM1 was fused to the C-terminus of *Ao*LPMO9A and *Ao*LPMO9B, respectively; (2) *Ao*LPMO9C∆CBM and *Nc*LPMO9C∆CBM, generated by truncating CBM1 from *Ao*LPMO9C and *Nc*LPMO9C, respectively; and (3) *Nc*LPMO9C∆L10/∆L30/∆L50, created by removing 10, 30, or 50 amino acids from the 79-amino-acid linker region of *Nc*LPMO9C (Figure 1). The gene fragments for *Ao*LPMO9C∆CBM and *Nc*LPMO9C∆CBM were amplified directly by PCR using the universal primer 5′ AOX and specific primers designed for each protein variant. The gene fragments for *Ao*LPMO9A+CBM, *Ao*LPMO9B+CBM, and *Nc*LPMO9C∆L10/∆L30/∆L50 were constructed using overlap PCR. First, downstream fragments containing CBM1 and part of the linker sequence, along with upstream fragments containing the catalytic domain and part of the linker sequence, were amplified by PCR with corresponding primers. The complete gene sequences for the variants were then assembled by fusing the upstream and downstream fragments using overlap PCR. The corresponding primer sequences used for variant construction are listed in Table S1.

### 4.4. Expression and Purification of AA9 LPMO Variants and AfCDH

The constructed DNA fragments were ligated into the pPICZαA vector, treated with the restriction enzymes *Bst*BI and *Eco*RI, to create the expression vectors pPICZαA-LPMOs. In these vectors, the α-factor sequence was replaced with the natural signal sequences. The recombinant vectors were linearized using the SacI restriction enzyme (NEB) and introduced into *Pichia pastoris* X33 via electroporation using a BIO-RAD electroporator (Hercules, CA, USA). The expression of wild-type AA9 LPMOs, including *Ao*LPMO9A, 9B, 9C, and *Nc*LPMO9C, as well as the constructed variants, followed previously established protocols [41]. All recombinant proteins were purified using Ni-NTA affinity chromatography [41] and incubated in a 100 mM sodium acetate solution (pH 5.0) containing 1 mM CuSO_4_ for copper (II) saturation at room temperature for 30 min. Excess free copper ions were removed by dialysis in sodium acetate buffer (pH 5.0) for 24 h, with buffer changes occurring three times to ensure the removal of free Cu^2^⁺ [41]. *Af*CDH from *A. fumigatus* was produced using recombinant *P. pastoris* as described in previous studies [56,57]. The purity of recombinant proteins was confirmed using sodium dodecyl sulfate-polyacrylamide gel electrophoresis (SDS-PAGE) with a 12% gel concentration. Protein concentrations were measured using a BCA kit (Thermo Fisher Scientific, Waltham, MA, USA).

### 4.5. The Oxidase Activity Assay

The oxidase activities of AA9 LPMOs and their variants were determined by measuring their H_2_O_2_ generation capacity using the horseradish peroxidase (HRP)/Amplex Red assay method [13,17,58]. A reaction mixture (3 mL) containing 1 µM of each AA9 LPMO, 15 units of HRP, and 100 µM Amplex Red in 50 mM sodium acetate buffer (pH 5.0) was pre-incubated for 5 min at 45 °C in a standard glass cuvette using a UV-2800 ultraviolet–visible spectrophotometer (Unico Instrument Co., Ltd., Shanghai, China). The reaction was initiated by adding AscA to a final concentration of 1 mM, and H_2_O_2_ production was monitored continuously by measuring the formation of resorufin at 563 nm for 30 min. Control reactions, in which AA9 LPMOs were replaced with ultrapure water or third-dialysate, were conducted in parallel to assess the rate of H_2_O_2_ production independent of AA9 LPMOs. All assays were performed in triplicate.

### 4.6. The Peroxidase Activity Assay

The peroxidase activity of wild-type AA9 LPMOs and their variants was measured using 2,6-dimethoxyphenol (2,6-DMP) as the substrate, which is oxidized to yellow coerulignone in the presence of H_2_O_2_ [59]. The reaction mixture (0.5 mL) contained 5 mM 2,6-DMP as the substrate, 100 µM H_2_O_2_, and 1 µM of enzyme in 100 mM sodium phosphate buffer (pH 6.0). The reaction was carried out at 37 °C for 5 min, and the absorbance was measured at 469 nm (ε469 = 53,200 M^−1^ cm^−1^). The amount of coerulignone produced was calculated based on the absorbance values. A control reaction was conducted under the same conditions, except the enzyme was replaced with third-dialysate, to assess the impact of residual free copper ions in the enzyme solution on peroxidase activity. One unit of enzyme activity was defined as the amount of enzyme that produces 1 µmol of coerulignone per minute.

According to the mechanism of the peroxidase reaction, AA9 LPMOs oxidize 2,6-DMP with the help of H_2_O_2_ as a co-substrate. Therefore, the kinetic constants of wild-type AA9 LPMOs and their variants were determined for both 2,6-DMP and H_2_O_2_. The kinetic constants for 2,6-DMP were determined using a reaction mixture (0.5 mL) containing various concentrations of 2,6-DMP (0–10 mM), 1 µM of enzyme, and 5 mM H_2_O_2_ as the co-substrate in 100 mM sodium phosphate buffer (pH 6.0). The kinetic constants for H_2_O_2_ were determined using a reaction mixture (0.5 mL) containing various concentrations of H_2_O_2_ (0.1 mM to 15 mM), 1 µM of enzyme, and 5 mM 2,6-DMP as the substrate in 100 mM sodium phosphate buffer (pH 6.0). Both reactions were performed at 37 °C for 5 min, and the absorbance at 469 nm was used to calculate the amount of coerulignone produced [59]. All kinetic parameters were determined by fitting the data to the Michaelis–Menten equation using nonlinear regression in GraphPad Prism8 software (http://www.graphpad.com/prism/, accessed on 1 October 2024) to calculate the *K*_m_ and *k*_cat_ values for each enzyme.

### 4.7. Measurement of Adsorption Capacity

PASC-80% was used as the cellulosic substrate to assess the adsorption properties of eleven wild-type AA9 LPMOs and their variants with different modular structures. The reaction mixture consisted of PASC-80% (4 mg/mL) and varying concentrations of enzyme (0–40 µM) in 50 mM sodium acetate buffer (pH 5.0), with a final volume of 0.5 mL. The binding reactions were conducted in 2 mL centrifuge tubes, shaken constantly (150 rpm) at 4 °C for 30 min. After the reaction, the supernatant was collected by centrifugation at 10,000 rpm for 10 min at 4 °C, and the enzyme concentration in the supernatant was measured at A280 using an Eppendorf Biophotometer (Eppendorf, Hamburg, Germany). All experiments were performed in triplicate, and control samples, either without enzymes or cellulose, were included under the same conditions. The dissociation constant (*K*_d_, µM) and maximum binding capacity (*B*_Max_, µmol/g PASC) were calculated using nonlinear fitting in Prism8 software (GraphPad). The binding curve was fitted to the equation ($Y=B_{Max}*X/{(K}_{d}+X$), where Y represents the bound protein and X represents the free protein in the adsorbent–adsorbate complex [22].

### 4.8. Effects of CBM1 and Linker on Monooxygenase Activity of Wild-Type AA9 LPMOs and Variants Using AfCDH or AscA as Electron Donor

The aim of this study was to evaluate the impact of CBM1 and the linker region on the monooxygenase activity of wild-type AA9 LPMOs and their variants during oxidation reactions with different types and concentrations of electron donors and various cellulose crystallinities. Reactions were carried out in 10 mL tubes containing 4 mg/mL of cellulosic substrate (PASC-80%, PASC-75%, or Avicel) and 1 µM of each AA9 LPMO enzyme in 2 mL of sodium acetate buffer (50 mM, pH 5.0). When AscA was used as the electron donor, either 0.2 mM or 1 mM AscA was added to the reaction system. When *Af*CDH from *A. fumigatus Af*293 was the electron donor, 0.06 µM or 0.3 µM *Af*CDH was added, along with 10 mM lactose. After incubation at 45 °C and 200 rpm for 24 h, the reactions were stopped by boiling at 99 °C for 10 min, followed by centrifugation at 10,000 rpm for 10 min. The supernatant was then analyzed by high-performance anion exchange chromatography with pulsed amperometric detection (HPAEC-PAD) according to previously described methods [41,60]. The HPAEC-PAD system was equipped with a PA200 separation column. The mobile phase consisted of 0.1 M NaOH and sodium acetate, with an elution flow rate of 0.4 mL/min. The sodium acetate concentration was varied as follows: 0 to 140 mM over 14 min, 140 to 300 mM over 8 min, 300 to 400 mM over 4 min, and 500 mM for 3 min.

### 4.9. H_2_O_2_ Concentration Measurement

The apparent H_2_O_2_ concentration in the supernatants after various time points during the oxidation reaction between AA9 LPMOs and the cellulosic substrate, as described in Section 2.5, was measured using the modified HRP/Amplex Red assay method. At specified time intervals, samples were collected via centrifugation. Subsequently, 50 µL of the supernatant was mixed with 50 µL of a freshly prepared premix containing HRP (10 U/mL) and Amplex Red (200 µM) in sodium phosphate buffer (50 mM, pH 6.0). The reaction mixture (100 µL) was incubated in a 96-well microtiter plate for 1 min before recording the absorbance at 563 nm. All assays were performed in triplicate. Control reactions, where AA9 LPMOs were replaced with ultrapure water, were conducted in parallel to evaluate H_2_O_2_ production independent of AA9 LPMOs. Standard solutions of H_2_O_2_ (0–100 µM) were prepared in 50 mM sodium acetate buffer (pH 5.0), with 1 mM AscA. H_2_O_2_ levels were calculated using a calibration curve generated from the H_2_O_2_ standards.

### 4.10. Statistical Analysis

All experiments were performed in triplicate and averaged. Multiple *t*-tests were used to determine whether significant differences existed between two sample groups. The *p* value obtained from the multiple *t*-test analysis indicated the significance of the difference between the observed data and the null hypothesis. A *p* value of less than 0.05, as calculated using GraphPad Prism 7, was considered to indicate a statistically significant difference between the two groups. Significance levels were indicated as follows: * *p* < 0.05, ** *p* < 0.01, *** *p* < 0.001, **** *p* < 0.0001.

## 5. Conclusions

This study demonstrates that CBM1 promotes the binding affinity of modular AA9 LPMOs to cellulose, influencing the oxidative reaction in ways that can either promote or hinder activity, depending on the reductant type and cellulose crystallinity. Generally, CBM1 was advantageous for the oxidative reaction on high-, medium-, and low-crystallinity celluloses when AscA was used as the electron donor. While CBM1 also promoted the oxidative reaction on high-crystallinity cellulose with *Af*CDH as the electron donor, it significantly impeded the apparent monooxygenase activity on low-crystallinity cellulose, such as PASC-80%, in the *Af*CDH-AA9 LPMO system. In this case, strong binding by CBM1 may limit electron transfer and H_2_O_2_ production, both of which are potentially rate-limiting factors. Linker truncation moderately to strongly affected binding affinity, oxidase, and peroxidase activity. However, these alterations had less impact on the apparent monooxygenase activity in the AscA-AA9 LPMO and *Af*CDH-AA9 LPMO systems compared to CBM1. These findings provide new insights into the functional significance of the diverse modular structures of AA9 LPMOs in fungi. Our work suggests that the multiplicity of AA9 LPMOs may be essential for fungi to adapt to varying types of available reductants and changes in the structural composition of carbohydrate polymers during different growth stages.

## Figures and Tables

**Table 1:** Initial H_2_O_2_ generation rates of AA9 LPMOs and their variants.

Enzymes	H_2_O_2_ Generation Rate (min^−1^)	Enzymes	H_2_O_2_ Generation Rate (µM/min/µmol)
*Ao*LPMO9A	500.0 ± 3.3	*Nc*LPMO9C∆CBM	170.0 ± 10.0
*Ao*LPMO9A+CBM	456.7 ± 10.0	*Nc*LPMO9C∆L10	100.0 ± 10.0
*Ao*LPMO9B	510.0 ± 13.3	*Nc*LPMO9C∆L30	93.3 ± 3.3
*Ao*LPMO9B+CBM	320.0 ± 10.0	*Nc*LPMO9C∆L50	80.0 ± 3.3
*Ao*LPMO9C	106.7 ± 6.7	AscA	33.3 ± 6.7
*Ao*LPMO9C∆CBM	243.3 ± 10.0	AscA + dialysate	33.3 ± 3.3
*Nc*LPMO9C	150.0 ± 16.7		

**Table 2:** Kinetic constants of peroxidase activity of wild-type AA9 LPMOs and their variants for 2,6-DMP and H_2_O_2._

	2,6-DMP			H_2_O_2_	
Enzyme	*K*_m_ (mM)	*k*_cat_ (s^−1^)	Enzyme	*K*_m_ (mM)	*k*_cat_ (s^−1^)
*Ao*LPMO9A	1.76 ± 0.31	1.48 ± 0.09	*Ao*LPMO9A	1.23 ± 0.11	1.83 ± 0.04
*Ao*LPMO9A+CBM	1.58 ± 0.22	1.58 ± 0.07	*Ao*LPMO9A+CBM	1.12 ± 0.09	2.04 ± 0.05
*Ao*LPMO9B	1.55 ± 0.14	1.33 ± 0.04	*Ao*LPMO9B	0.81 ± 0.13	1.83 ± 0.08
*Ao*LPMO9B+CBM	1.16 ± 0.16	1.02 ± 0.04	*Ao*LPMO9B+CBM	0.88 ± 0.09	1.69 ± 0.04
*Ao*LPMO9C	0.46 ± 0.07	0.07 ± 0.002	*Ao*LPMO9C	1.13 ± 0.31	0.23 ± 0.02
*Ao*LPMO9C∆CBM	0.40 ± 0.05	0.09 ± 0.002	*Ao*LPMO9C∆CBM	0.23 ± 0.08	0.28 ± 0.02
*Nc*LPMO9C	0.94 ± 0.09	0.58 ± 0.02	*Nc*LPMO9C	0.17 ± 0.05	0.47 ± 0.01
*Nc*LPMO9C∆CBM	1.28 ± 0.12	0.53 ± 0.01	*Nc*LPMO9C∆CBM	0.15 ± 0.05	0.34 ± 0.02
*Nc*LPMO9C∆L10	0.64 ± 0.11	0.39 ± 0.02	*Nc*LPMO9C∆L10	0.37 ± 0.11	0.33 ± 0.02
*Nc*LPMO9C∆L30	0.87 ± 0.14	0.29 ± 0.01	*Nc*LPMO9C∆L30	0.31 ± 0.08	0.33 ± 0.02
*Nc*LPMO9C∆L50	0.72 ± 0.10	0.41 ± 0.02	*Nc*LPMO9C∆L50	0.36 ± 0.12	0.32 ± 0.02

**Table 3:** The binding properties of wild-type AA9 LPMOs and variants on 80%-PASC.

Enzymes	*B*_Max_ (μmol/g PASC)	*K*_d_ (μM)	Enzymes	*B*_Max_ (μmol/g PASC)	*K*_d_ (μM)
*Ao*LPMO9A	1.51 ± 0.53	8.84 ± 6.42	*Nc*LPMO9C	2.34 ± 0.34	1.64 ± 0.64
*Ao*LPMO9A+CBM	3.99 ± 0.17	1.01 ± 0.22	*Nc*LPMO9C∆CBM	0.43 ± 0.13	1.76 ± 1.12
*Ao*LPMO9B	0.48 ± 0.13	6.09 ± 3.32	*Nc*LPMO9C∆L10	2.69 ± 0.21	1.36 ± 0.57
*Ao*LPMO9B+CBM	2.46 ± 0.28	0.35 ± 0.21	*Nc*LPMO9C∆L30	4.56 ± 0.32	1.54 ± 0.43
*Ao*LPMO9C	4.07 ± 0.46	0.78 ± 0.43	*Nc*LPMO9C∆L50	4.85 ± 0.22	0.89 ± 0.27
*Ao*LPMO9C∆CBM	1.65 ± 0.62	16.68 ± 9.47			

## Data Availability

The original contributions presented in this study are included in the article/Supplementary Material. Further inquiries can be directed to the corresponding author.

## Supplementary Materials

The following supporting information can be downloaded at https://www.mdpi.com/article/10.3390/ijms252312616/s1.

## Abbreviations

CBM	Carbohydrate-binding module
AA9	Auxiliary activity 9
LPMO	Lytic polysaccharide monooxygenase
AscA	Ascorbic acid
CDH	Cellobiose dehydrogenase
GH	Glycoside hydrolase
CD	Catalytic domain
H_2_O_2_	Hydrogen peroxide
2,6-DMP	2,6-dimethoxyphenol
DP	Degrees of polymerization
PASC	Phosphoric acid-swollen cellulose
PASC-80%	PASC by swelling with 80% phosphoric acid
NGS	N-glycosylation site
CrI	Crystallinity index
SDS-PAGE	Sodium dodecyl sulfate-polyacrylamide gel electrophoresis
HRP	Horseradish peroxidase
HPAEC-PAD	High-performance anion exchange chromatography with pulsed amperometric detection
