# Supplementary material for: The Effect of CBM1 and Linker on the Oxidase, Peroxidase and Monooxygenase Activities of AA9 LPMOs: Insight into Their Correlation with the Nature of Reductants and Crystallinity of Celluloses
Source: Int J Mol Sci. 2024 Nov 24;25(23):12616. doi: 10.3390/ijms252312616 (PMC11641334; doi:10.3390/ijms252312616)
Supplement: Supplementary file 1 [file ijms-25-12616-s001.zip › ijms-3275448-supplementary.pdf]

The Effect of CBM1 and Linker on the Oxidase, Peroxidase and Monooxygenase Activities of AA9 LPMOs: Insight into their Correlation with the Nature of Reductants and Crystallinity

Xu Zhao, Fei Xie, Kaixiang Chen, Liangkun Long, Shaojun Ding*

The Co-Innovation Center of Efficient Processing and Utilization of Forest Resources, Jiangsu Key Lab for the Chemistry & Utilization of Agricultural and Forest Biomass, College of Chemical Engineering, Nanjing Forestry University, Nanjing 210037, Jiangsu, China

* Corresponding author:

Dr. Shaojun Ding,

College of Chemical Engineering, Nanjing Forestry University, Nanjing, Jiangsu 210037, China

Tel: +86 25 85427939

Fax: +86 25 85418873

E-mail: dshaojun@hotmail.com, dshaojun@njfu.edu.cn

Table S1. Primers designed for the construction of all AA9 LPMOs variants (The upstream primer and primer 1 are used for amplification of upstream fragment, and the primer 2 and downstream primer are used for amplification for the downstream fragment)

Genes	Primer sequences
AoLPMO9A+CBM	Upstream: 5′ -GACTGGTTCCAATTGACAAGC-3′ (5′ AOX)<br>Primer 1: 5′ -ACCACCAGAACCACCAGCAGGAGCAGAGAAAACCTCTGGACC-3′<br>Primer 2: 5′ -GGTCCAGAGGTTTTCTCTGCTCCTGCTGGTGGTTCTGGTGGT -3′<br>Downstream: 5′ -GCAAATGGCATTCTGACATCC-3′ (3′ AOX)
*Ao*LPMO9B+CBM	Upstream: 5′ -GACTGGTTCCAATTGACAAGC-3′ (5′ AOX)<br>Primer 1: 5′ -ACCGTACAAAGCAGCAGCTCTTCTTCTAGCCGCGGCAGAAGA-3′<br>Primer 2: 5′ -TCTTCTGCCGCGGCTAGAAGAAGAGCTGCTGCTTTGTACGGT-3′<br>Downstream: 5′ -GCAAATGGCATTCTGACATCC-3′ (3′ AOX)
*Ao*LPMO9CΔCBM	Upstream: 5′ -GACTGGTTCCAATTGACAAGC-3′ (5′ AOX)<br>Downstream: 5′ -CCGGAATTCTTAGTGATGGTGATGGTGATGACCAGCATTAGAAGATGGTTG-3′
*Nc*LPMO9CΔCBM	Upstream: 5′ -GACTGGTTCCAATTGACAAGC-3′ (5′ AOX)<br>Downstream: 5′ -CCGGAATTCTTAGTGATGGTGATGGTGATGAACAGCAGAGTTACCACCATT-3′
*Nc*LPMO9CΔL10	Upstream: 5′ -GACTGGTTCCAATTGACAAGC-3′ (5′ AOX)<br>Primer 1: 5′ -AGTAGTAGCAGCTGGTTTAGAAGTACCAGAACCAGAACCATT-3′<br>Primer 2: 5′ -AATGGTTCTGGTTCTGGTACTTCTAAACCAGCTGCTACTACT-3′<br>Downstream: 5′ -GCAAATGGCATTCTGACATCC-3′ (3′ AOX)
*Nc*LPMO9CΔL30	Upstream: 5′ -GACTGGTTCCAATTGACAAGC-3′ (5′ AOX)<br>Primer 1: 5′ -ACCATTAGTAGGTTGTGGGTTTGGAGTGGAGGCGGTGGTAGA-3′<br>Primer 2: 5′ -TCTACCACCGCCTCCACTCCAAACCCACAACCTACTAATGGT-3′<br>Downstream: 5′ -GCAAATGGCATTCTGACATCC-3′ (3′ AOX)
*Nc*LPMO9CΔL50	Upstream: 5′ -GACTGGTTCCAATTGACAAGC-3′ (5′ AOX)<br>Primer 1: 5′ -CAAAGCAGCAGCTCTAACAGCTGGAGCAGAAGCAGAAGTAGG-3′<br>Primer 2: 5′ -CCTACTTCTGCTTCTGCTCCAGCTGTTAGAGCTGCTGCTTTG-3′<br>Downstream: 5′ -GCAAATGGCATTCTGACATCC-3′ (3′ AOX)

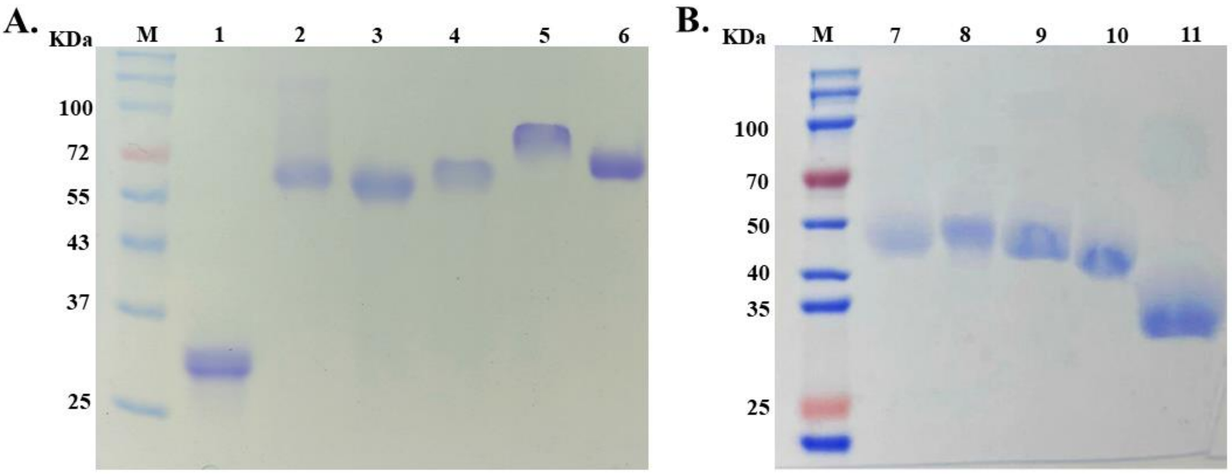

Figure S1. SDS-PAGE analysis of recombinant wild-type AA9 LPMOs and variants. lane M, Marker; Lane 1-2, *Ao*LPM09A and *Ao*LPMO9A+CBM; Lane 3-4 *Ao*LPMO9B and *Ao*LPMO9B+CBM; Lane 5-6, *Ao*LPMO9C and *Ao*LPMO9CΔCBM; Lane 7-11, *Nc*LPMO9C, *Nc*LPMO9CΔCBM, *Nc*LPMO9CΔL10, *Nc*LPMO9CΔL30, and *Nc*LPMO9CΔL50, respectively.

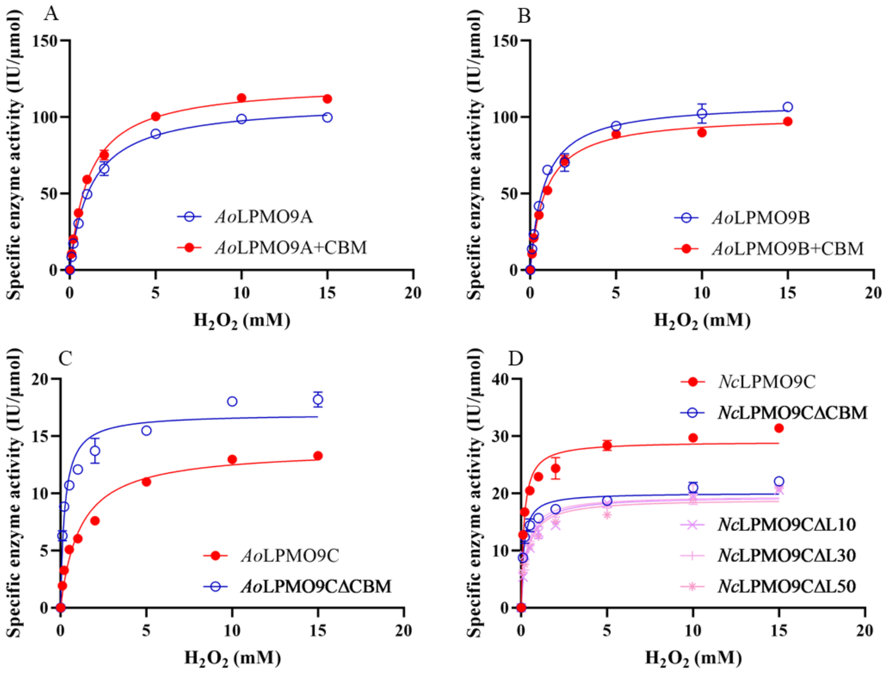

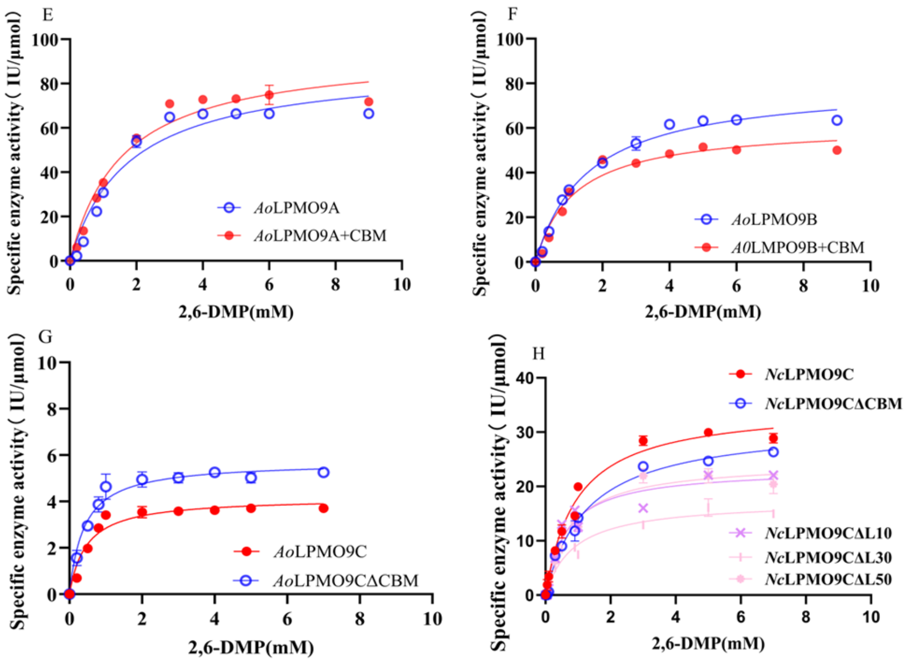

Figure S2. Michaelis–Menten kinetics of wild-type AA9 LPMOs and variants for H_2O_2 and 2,6-DMP, respectively. A-D: The peroxidase activity of wild-type AA9 LPMOs and variants was measured at different H_2O_2 concentrations (0–15 mM) as co-substrate with 5.0 mM 2, 6-DMP as substrate. A: *Ao*LPMO9A and its CBM1-fused variant B: *Ao*LPMO9B and its CBM1-fused variant; C: *Ao*LPMO9C and its CBM1-truncated variant; D: *Nc*LPMO9C and its CBM1- or linker-truncated variants. E-H: The peroxidase activity of wild-type AA9 LPMOs and variants was measured at different 2,6-DMP concentrations (0–10 mM) as substrate with 5.0 mM H_2O_2 as co-substrate. E: *Ao*LPMO9A and its CBM1-fused variant F: *Ao*LPMO9B and its CBM1-fused variant; G: *Ao*LPMO9C and its CBM1-truncated variant; H: *Nc*LPMO9C and its CBM1- or linker-truncated variants. No peroxidase activity was detected in the control reaction in which the enzyme was replaced with third-dialysate, so the data of controls are not shown in the figure. All the assays were performed in triplicate.

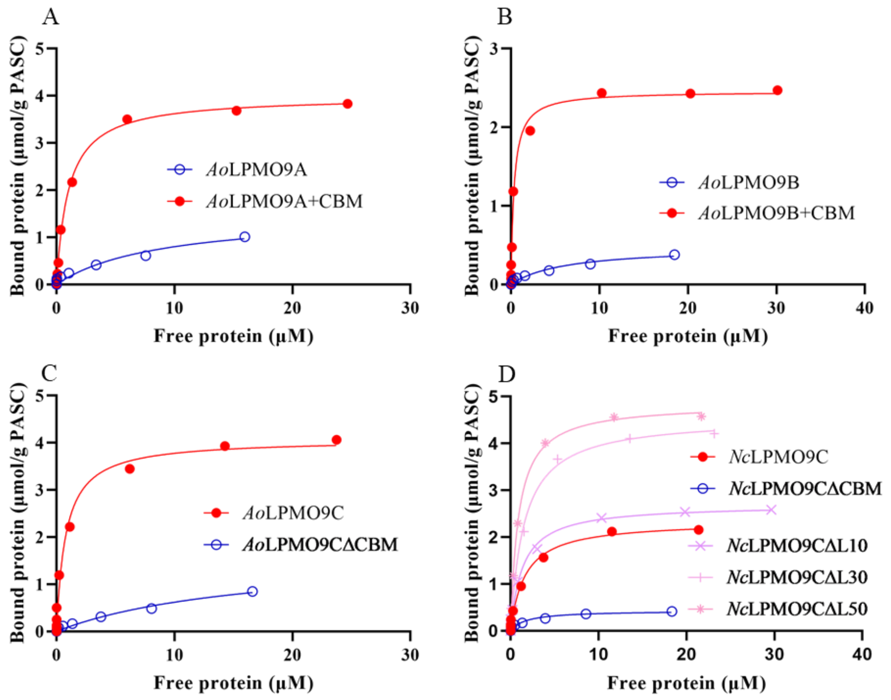

Figure S3. Binding isotherms for wild-type AA9 LPMOs and variants on 80% PASC. A: *Ao*LPMO9A and its CBM1-fused variant B: *Ao*LPMO9B and its CBM1-fused variant; C: *Ao*LPMO9C and its CBM1-truncated variant; D: *Nc*LPMO9C and its CBM1- or linker-truncated variants. All the assays were performed in triplicate.

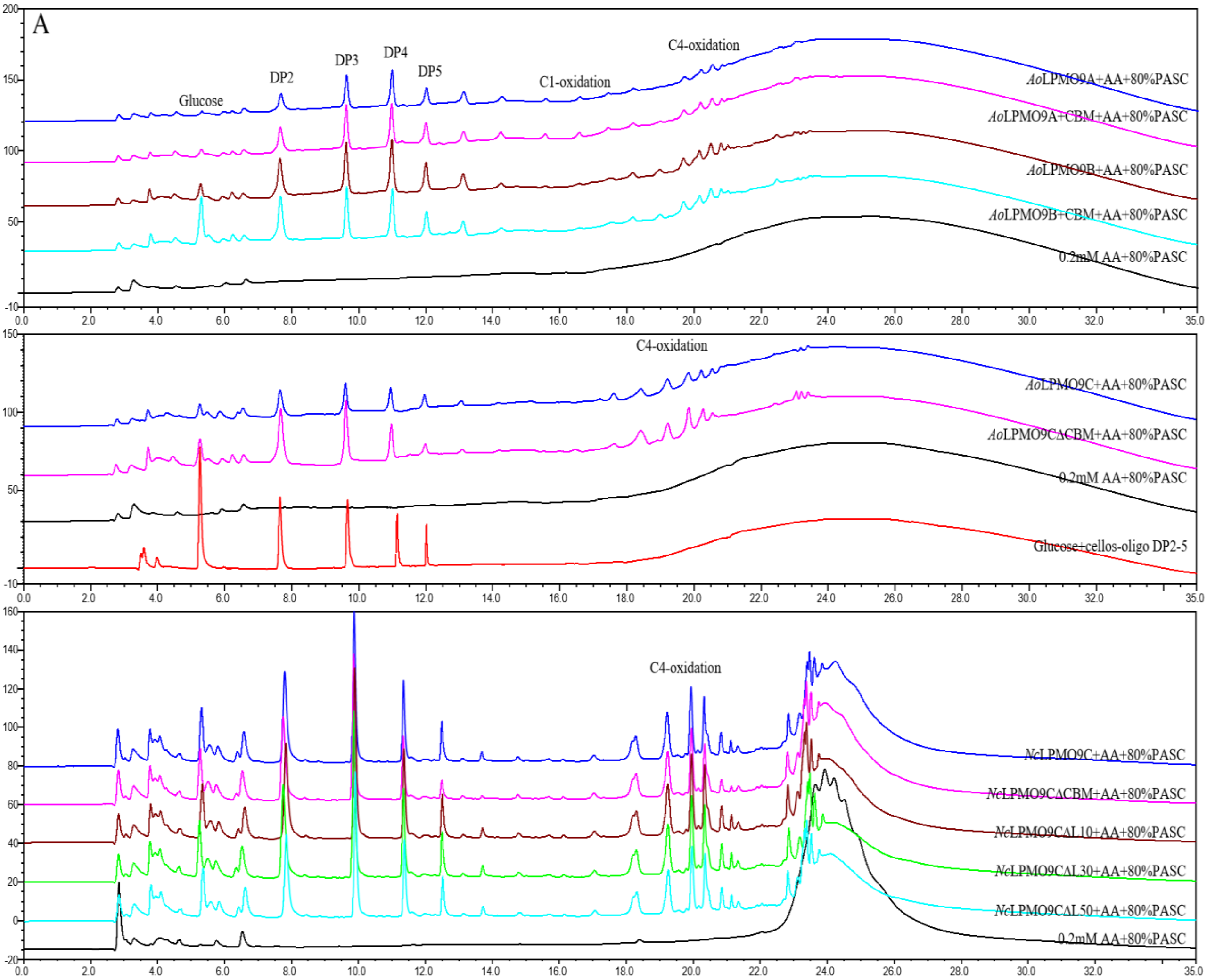

A
Glucose
DP2
DP3
DP4
DP5
C1-oxidation
C4-oxidation
AoLPMO9A+AA+80%PASC
AoLPMO9A+CBM+AA+80%PASC
AoLPMO9B+AA+80%PASC
AoLPMO9B+CBM+AA+80%PASC
0.2mM AA+80%PASC
C4-oxidation
AoLPMO9C+AA+80%PASC
AoLPMO9CΔCBM+AA+80%PASC
0.2mM AA+80%PASC
Glucose+cellos-oligo DP2-5
C4-oxidation
NcLPMO9C+AA+80%PASC
NcLPMO9CΔCBM+AA+80%PASC
NcLPMO9CΔL10+AA+80%PASC
NcLPMO9CΔL30+AA+80%PASC
NcLPMO9CΔL50+AA+80%PASC
0.2mM AA+80%PASC

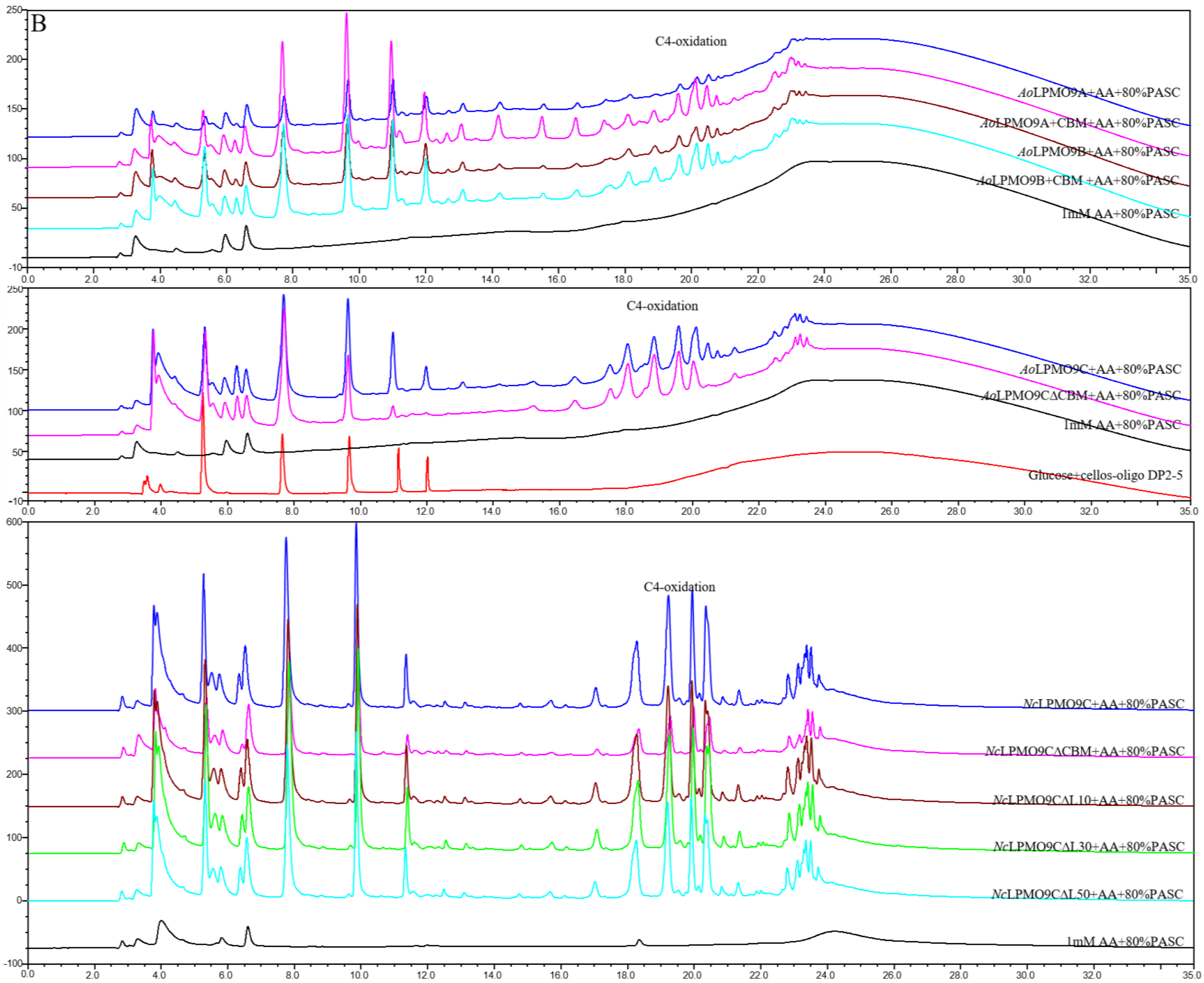

Figure S4. HPAEC-PAD analysis for AA9 LPMO oxidative activity on 80% PASC substrate with 0.2 mM AA (A) or 1 mM AA (B) as electron donor

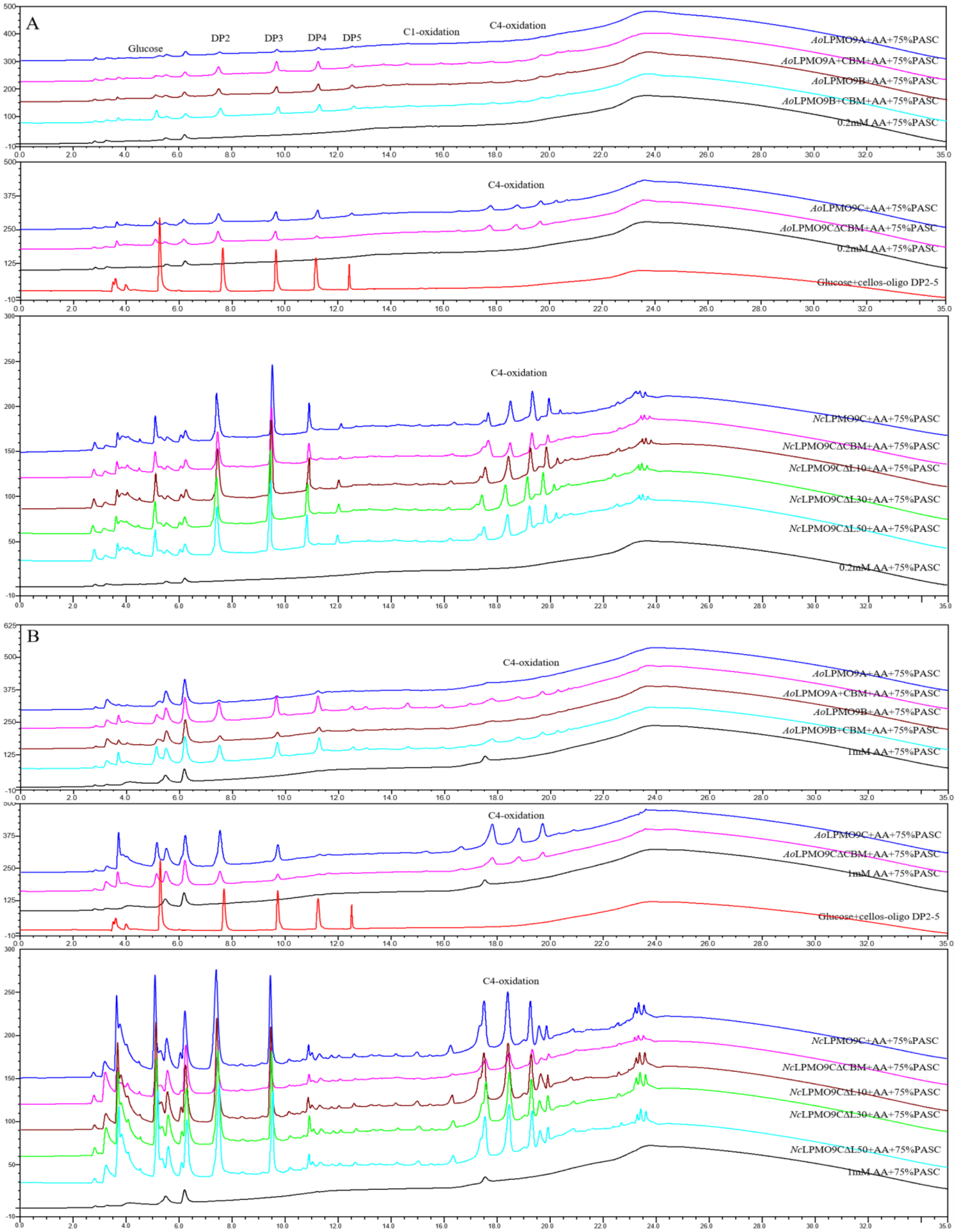

Figure S5. HPAEC-PAD analysis for AA9 LPMO oxidative activity on 75% PASC substrate with 0.2 mM AA (A) or 1 mM AA (B) as electron donor

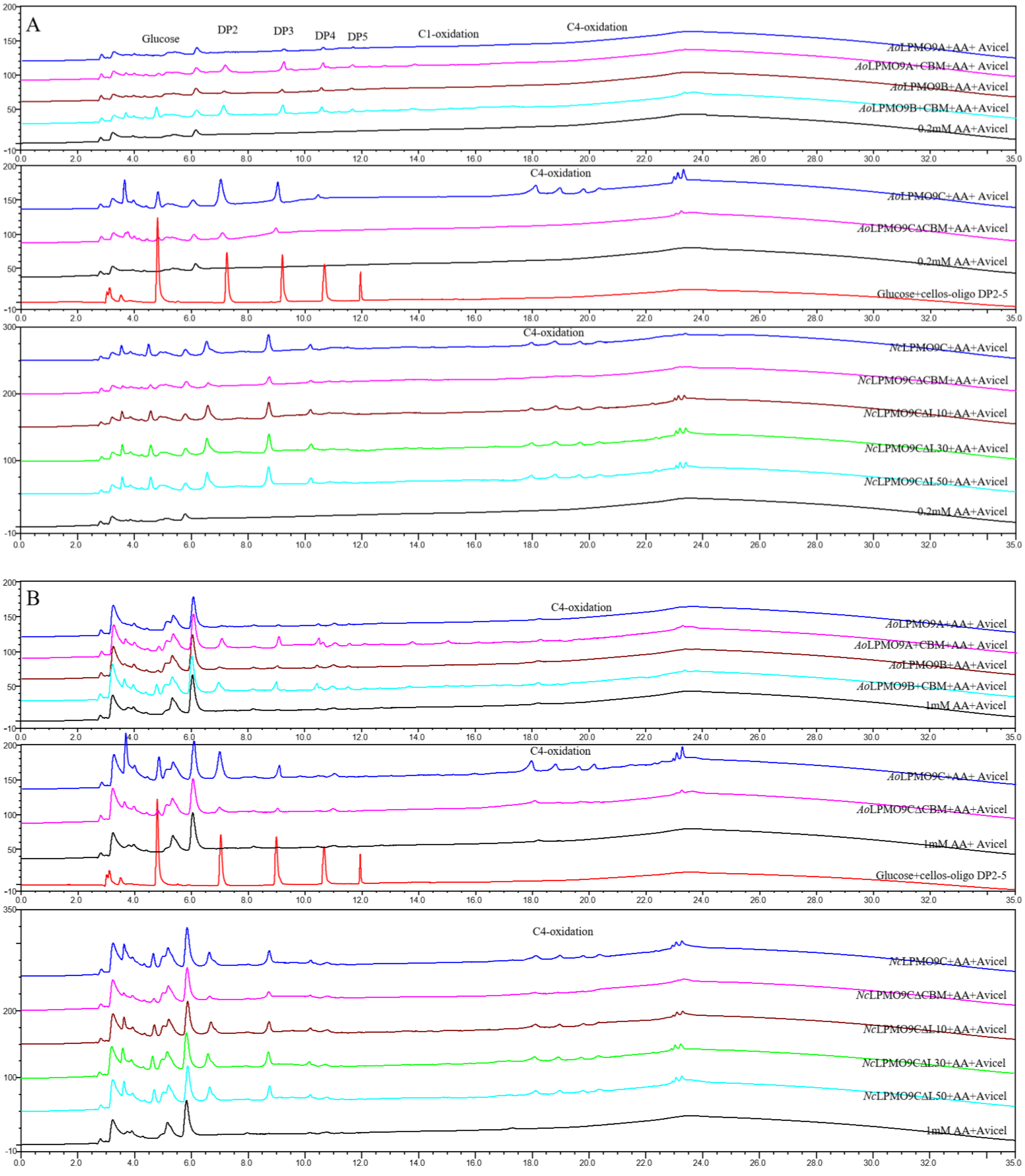

Figure S6. HPAEC-PAD analysis for AA9 LPMO oxidative activity on Avicel substrate with 0.2 mM AA (A) or 1 mM AA (B) as electron donor

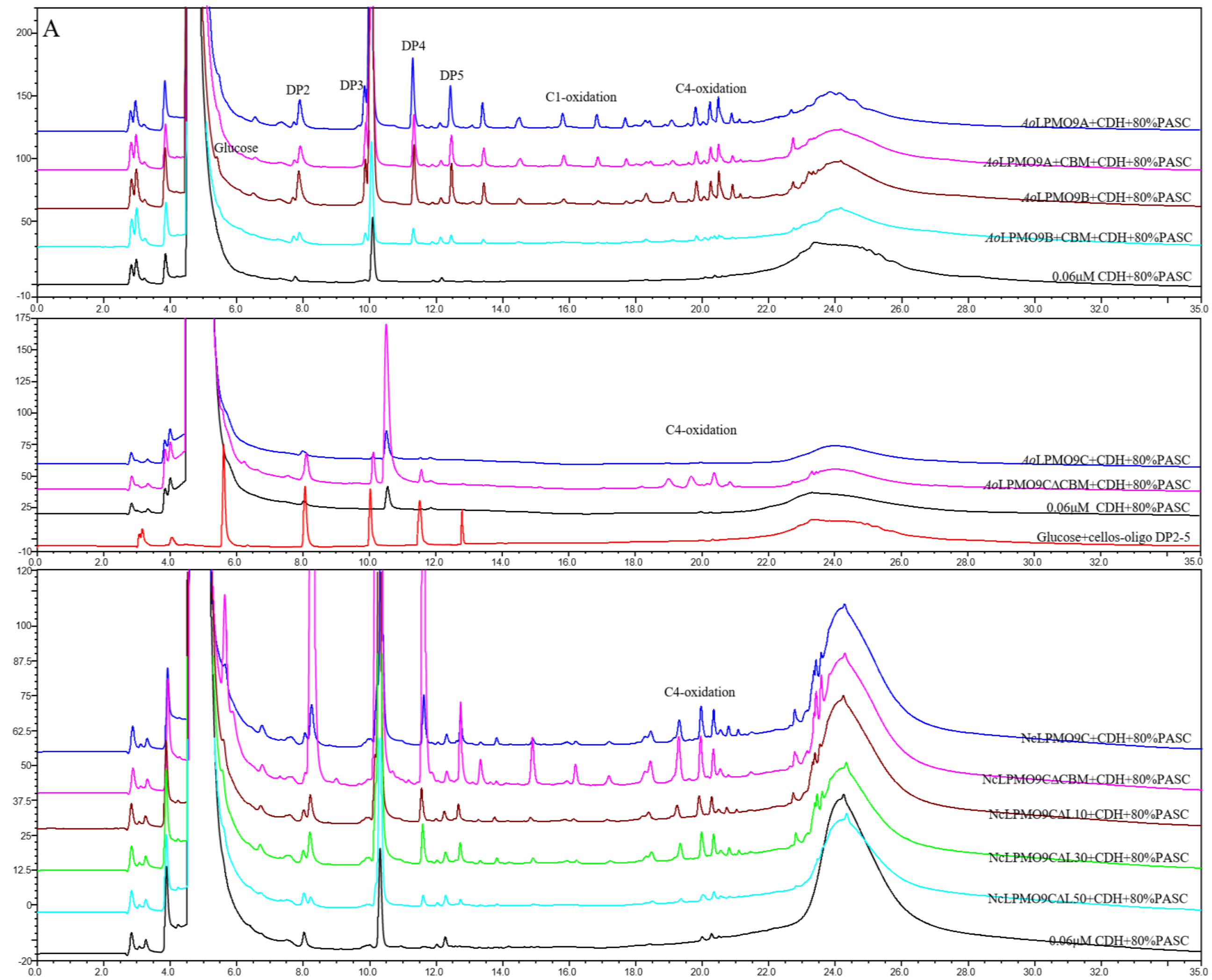
A
Glucose
DP2
DP3
DP4
DP5
C1-oxidation
C4-oxidation
AoLPMO9A+CDH+80%PASC
AoLPMO9A+CBM+CDH+80%PASC
AoLPMO9B+CDH+80%PASC
AoLPMO9B+CBM+CDH+80%PASC
0.06μM CDH+80%PASC
C4-oxidation
AoLPMO9C+CDH+80%PASC
AoLPMO9CΔCBM+CDH+80%PASC
0.06μM CDH+80%PASC
Glucose+cellos-oligo DP2-5
C4-oxidation
NcLPMO9C+CDH+80%PASC
NcLPMO9CΔCBM+CDH+80%PASC
NcLPMO9CΔL10+CDH+80%PASC
NcLPMO9CΔL30+CDH+80%PASC
NcLPMO9CΔL50+CDH+80%PASC
0.06μM CDH+80%PASC

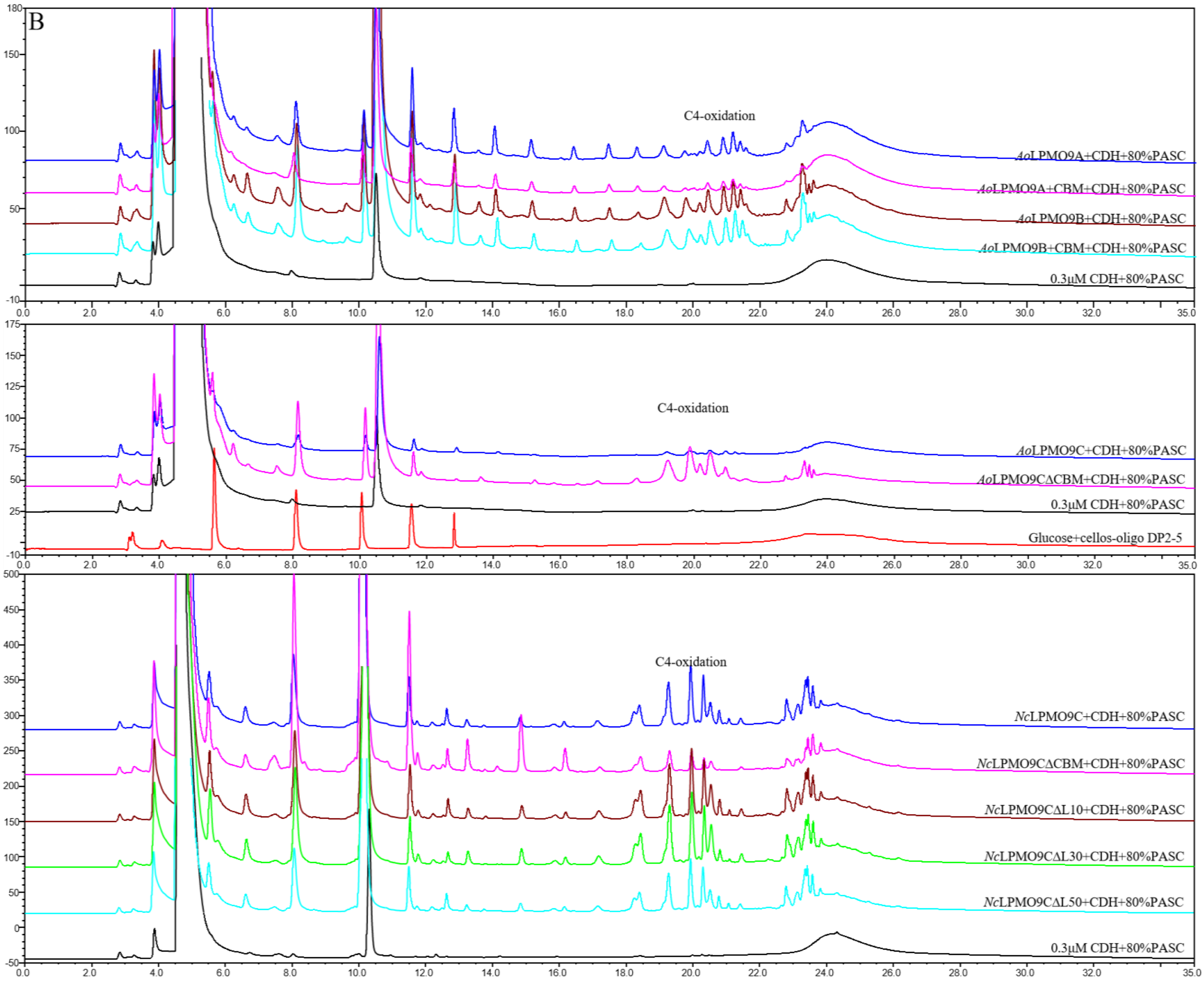

Figure S7. HPAEC-PAD analysis of AA9 LPMO oxidative activity on 80% PASC substrate with 0.06 μM CDH (A) or 0.3 μM CDH (B) as electron donor

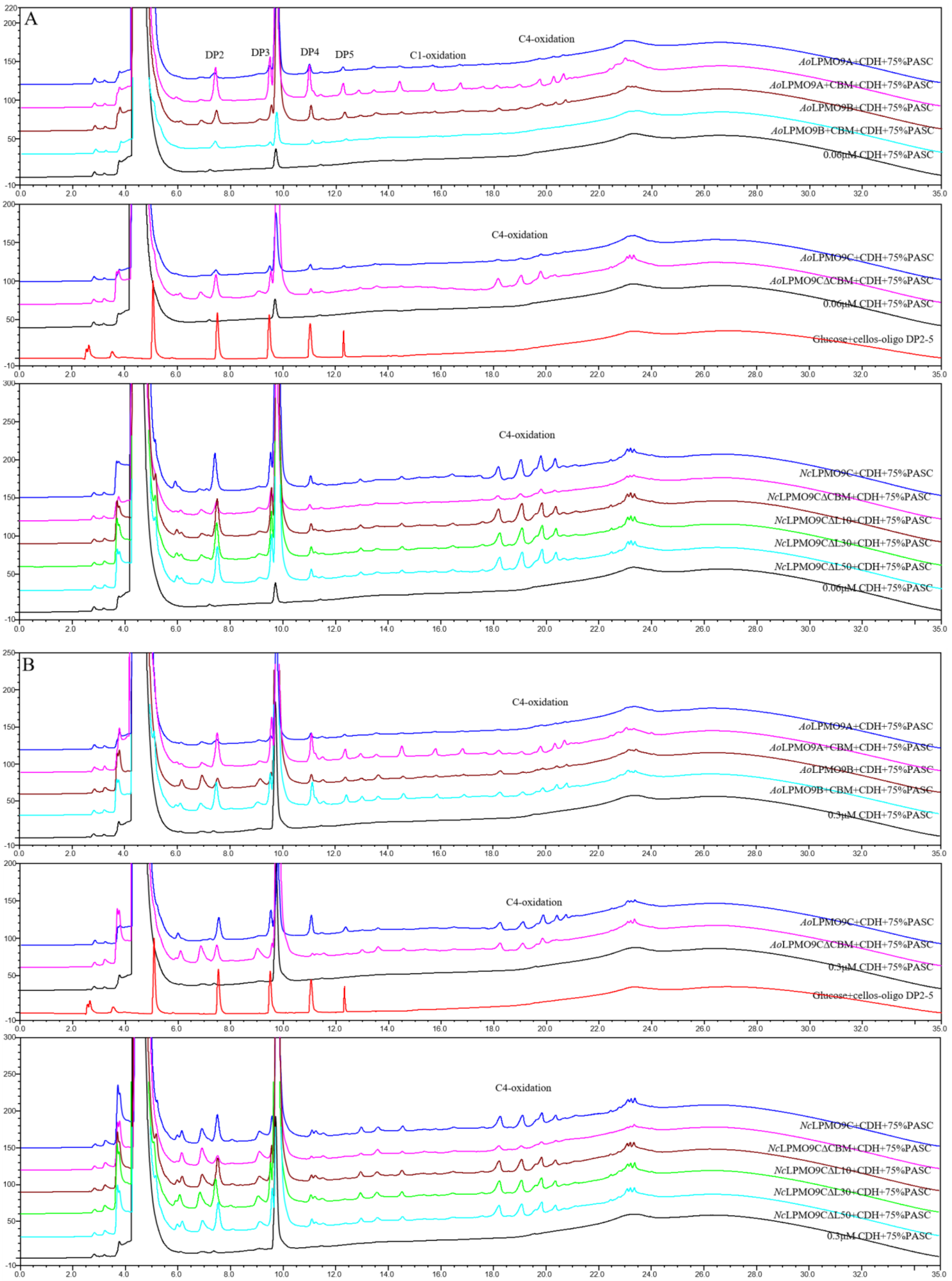

Figure S8. HPAEC-PAD analysis of AA9 LPMO oxidative activity on 75% PASC substrate with 0.06 μM CDH (A) or 0.3 μM CDH (B) as electron donor

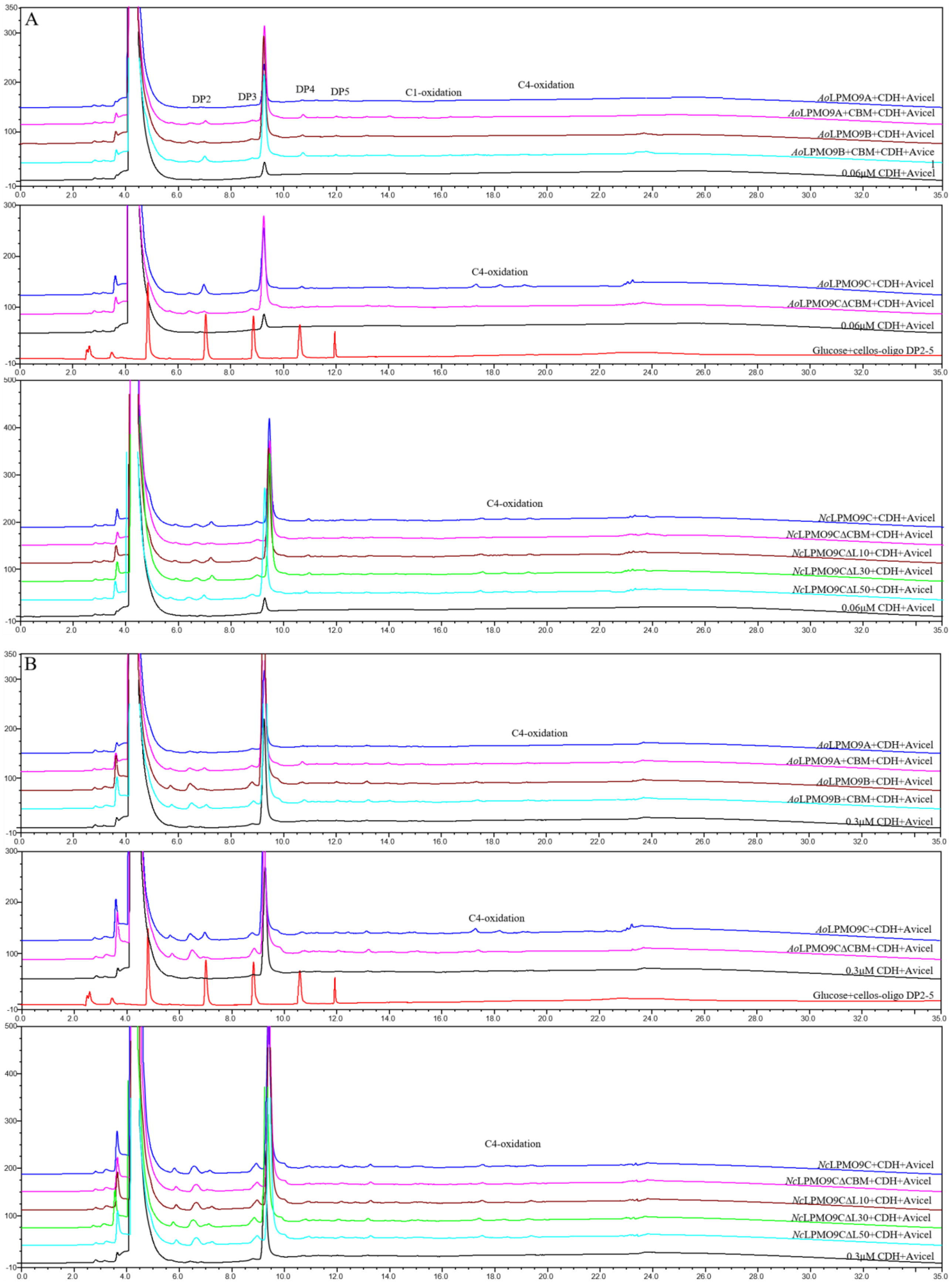

Figure S9. HPAEC-PAD analysis of AA9 LPMO oxidative activity on Avicel substrate with 0.06 μM CDH (A) or 0.3 μM CDH (B) as electron donor
